# Introducing Sulfur Ylides as Charge-Neutral Termini for Mitigating Poly(ethylene glycol) Antigenicity in Nanomedicine

**DOI:** 10.1021/jacsau.5c00748

**Published:** 2025-09-05

**Authors:** Dulce M. Sánchez-Cerrillo, Kouichi Shiraishi, Lucía Mallen-Huertas, Remi Peters, Daniela A. Wilson, Kevin Neumann

**Affiliations:** † Systems Chemistry Department, Institute for Molecules and Materials, 98810 Radboud University Nijmegen, Heyendaalseweg 135, Nijmegen 6525 AJ, The Netherlands; ‡ Research Center for Medical Sciences, the Jikei University School of Medicine, Minato-ku, Tokyo 105-8461, Japan

**Keywords:** PEGylation, antibody recognition, sulfur ylides antigenicity, nanomedicine

## Abstract

The widespread use of polyethylene glycol (PEG) in biomedical applications has led to the emergence of anti-PEG antibodies, which accelerate systemic clearance and undermine the performance of PEGylated systems, including those of nanomedicines. Antibody recognition often involves the hydrophobic PEG terminus, highlighting the need for alternative end-functionalization strategies that enhance hydrophilicity while maintaining stealth properties. Here, we introduce a novel PEGylation concept using sulfur ylides bearing tri- and pentapeptides as terminal modifications. These ylide-PEG (yPEG) conjugates were integrated into polymeric nanoparticles as a model system, demonstrating that ylide functionalization maintains key physicochemical properties, such as ζ-potential and antifouling behavior. Crucially, antibody binding assays with monoclonal IgM and IgG anti-PEG antibodies revealed that the ylide terminus significantly reduces recognition by both main chain- and terminus-specific anti-PEG antibodies. Experiments with polyclonal anti-PEG antibodies from mPEG-immunized mice suggest that increasing the chemical complexity of the PEG terminus with a strongly hydrophilic yet overall charge-neutral group effectively prevents antigenicity from extending to the terminus, ultimately reducing PEG-specific recognition. This modular and scalable strategy of yPEGs offers a new paradigm for engineering stealth-functionalized polymers with broad implications for nanomedicine, biomaterials, and surface coatings.

## Graphical abstract

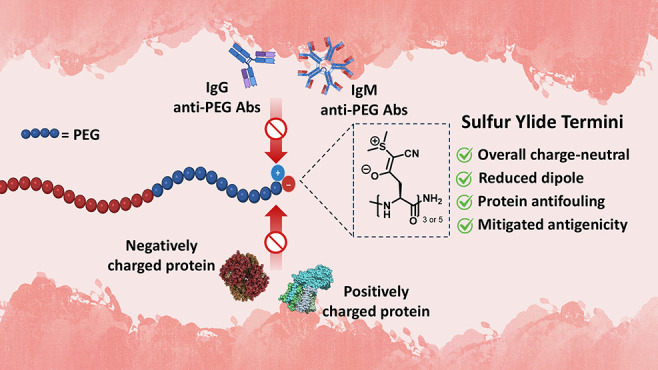

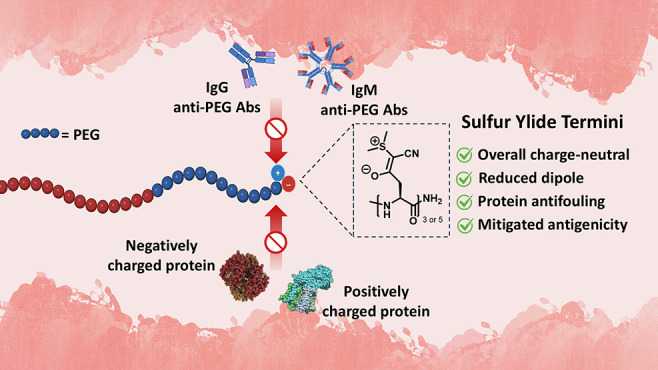

## Introduction

Polyethylene glycol (PEG) has been extensively used for nanomedical applications such as drug delivery over the last few decades. At least 43 medicines containing PEG in their formulations were approved by the European Medicines Agency (EMA). The stealth effect of PEG, along with its hydrophilicity, and biocompatibility, makes these systems suitable for nanomedical applications.^1–4^ However, the use of this polymer is not limited to pharmaceutical purposes; it is also widely used in formulations for daily-use products, e.g., cosmetics and health care products. The frequent use of this polymer has led to the development of anti-PEG antibodies (anti-PEG Abs) in healthy individuals.^5–9^ Such antibodies are responsible for the accelerated blood clearance (ABC) phenomenon, which presents a significant therapeutic challenge. In this process, anti-PEG Abs bind to PEGylated species, including nanocarriers, triggering their rapid elimination from the bloodstream upon repeated administration. This mechanism often involves activation of the complement system. The immune response can range from enhanced nanoparticle clearance to severe adverse effects, including anaphylaxis.^10–13^

Such outcomes not only compromise the therapeutic efficacy of PEGylated nanomedicines but also raise safety concerns, with the most recent example observed during the administration of mRNA vaccines against SARS-CoV-2, which utilize formulations incorporating PEGylated lipid nanoparticles.^14–16^ Some studies have suggested that the exposure of individuals to PEG could have promoted the development of antibodies and therefore caused more severe immunogenic reactions to the vaccine than in those without prior exposure to the polymer.^17^ Moreover, it was observed that vaccination with BNT162b2 (Pfizer-BioNTech) or mRNA-1273 (Moderna) significantly boosted the levels of both IgG and IgM PEG-specific antibodies.^18^ Additionally, recent studies have demonstrated that after vaccination with Moderna SPIKEVAX, the PEG immunogenicity is highly influenced by the circulation of mRNA lipid nanoparticles in the bloodstream as well as its clearance via phagocytosis.^19^ Further studies have also highlighted the effect of such antibodies present in the protein corona of nanocarriers, which enhances cellular uptake by macrophages, leading to the inefficiency of these therapies.^20^ The use of such particles is not limited to mRNA vaccines but also extends to other therapeutic applications such as chronic lymphocytic leukemia (CLL). Recently, it was demonstrated that the efficacy of CD20-targeted nanoparticles, used for CLL treatment and functionalized with PEG, is strongly influenced by the presence of anti-PEG antibodies. Patients with lower levels of these antibodies showed higher targeting efficiency.^21^

To overcome the challenges associated with the use of PEGylated systems, scientists and the pharmaceutical industry have focused on developing polymers that retain the beneficial properties of PEG including biocompatibility, hydrophilicity, and stealth effects. Examples include polyoxazolines,^22–25^ polypeptides,^26–29^ polyesters,^30^ glycopolymers,^31^ and zwitterionic polymers.^32–34^ Although several elegant examples have been reported that demonstrate the potential of these alternatives, transitioning to an industrial scale, as well as substituting PEGs in drug delivery formulations, may represent a significant challenge. This is because PEGylated systems have been extensively studied and optimized over the last decades. Alternatively, understanding the mechanism behind the binding of anti-PEG Abs to the polymeric chain may enable the design of alternative PEG derivatives that do not require large changes in the chemical structure or synthesis of this polymer but improve their performance in drug delivery, for example, by avoiding recognition by the body’s immune system. Further research to understand this phenomenon and the processes behind it has suggested how these antibodies can bind to PEG in different manners and activate the complement immune system. It has been noted that, in particular, the corresponding hydrophobic block in amphiphilic block copolymers plays a crucial role in recognition by anti-PEG Abs.^35^ For instance, the addition of an intermediate polyanionic block, consisting of poly­(aspartic acid) (poly­(Asp)), between PEG and poly­(l-phenylalanine) revealed a suppression of anti-PEG IgM binding. The authors attributed these findings to the possible electrostatic repulsion caused by the poly­(Asp) spacer.^36^ Another example is the incorporation of an intermediate hydrophilic block, such as poly­(sarcosine), between methoxy PEG (mPEG) and poly­(l-isoleucine) in which the binding affinity of anti-PEG Abs decreased compared to the block copolymer that contains only mPEG and poly­(l-isoleucine).^37^ Nevertheless, the binding affinity is not only influenced by the incorporation of large blocks as part of the block copolymer structure, but it has also been found that the relatively small terminus of PEG plays an important role and can facilitate binding to antibodies. For instance, after the administration of liposomes composed of PEG with methoxy, amine, carboxylic acid, or hydroxy termini, mPEG showed a higher production of anti-PEG immunoglobulin M (IgM), ultimately reducing the immunogenicity and antigenicity.^38^ However, the latter is more prone to biodegradation,^39,40^ while the introduction of charges directly impacts nonspecific protein adhesion via enhanced electrostatic interaction with blood plasma proteins.^41^ In addition, after repeated administration, the binding of IgM antibodies (Abs) to the liposomes could not be avoided, activating the complement system and subsequently leading to accelerated clearance.^38^ Consequently, there is an unmet need to identify terminal PEG groups that preserve stealth properties while exhibiting strong hydrophilic characteristics.

The design of efficient therapeutic nanocarriers that can also circumvent the formation of a protein corona to avoid immune cell interactions is a factor that should be considered. The result of this phenomenon could lead to inefficient targeting or accelerated clearance of the nanoparticles from the body. It has been demonstrated that the coating of nanoparticles has a strong influence on the way biomacromolecules interact with the surface.^42–44^ Zwitterions are well-known scaffolds for the design of hydrophilic materials including antifouling coatings.^45,46^ Polymeric zwitterionic structures are typically obtained by direct polymerization of corresponding monomers or via postpolymerization modification. Notably, compared to hydrophilic polyelectrolytes, zwitterionic materials are overall charge-neutral, further preventing nonspecific protein adsorption on the nanocarrier, making these compounds suitable for the efficient transport of therapeutics.^47–49^ It has been demonstrated that by reducing the intermolecular distance between the positive and negative charges, the nonfouling properties tend to increase,^50,51^ which recently led to the introduction of poly­(ylides)—a distinct class of hydrophilic polymers in which the positive charge is directly adjacent to the negative charge.^52,53^ While poly­(ylides) share many advantageous properties with zwitterionic polymers, their minimal dipole moment, salt resistance, and relatively small size set them apart from traditional betaine scaffolds.

Encouraged by these properties displayed by ylide residues, herein, we present for the first time the end-group modification of PEGylated polymers with ylide residues (Figure 1). We demonstrate that the conjugation of short ylide-containing peptides to amphiphilic poly­(ethylene glycol)-*block*-poly­(lactic-*co*-glycolic acid) (PEG*-b-*PLGA) copolymers enhances the nanoparticles’ characteristics, such as hydrophilicity, avoidance of particle aggregation, minimal change in zeta potential acr different pH values, and preventing nonspecific protein binding. These findings open new possibilities for functionalization of well-established PEGylated systems used for nanomedical applications without the need for altering the PEG backbone.

**Figure 1:**
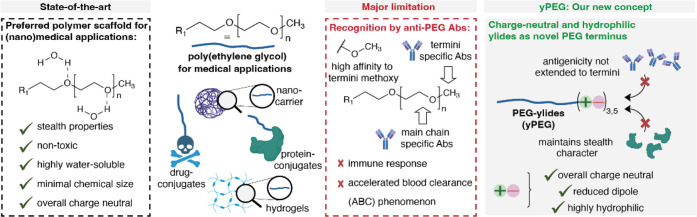
Schematic illustration of advantages and limitations of PEGylated systems in the context of (nano)­medicine. Ylides provide a hydrophilic terminus that maintains stealth characteristics while reducing the level of PEG-specific antibody recognition. The figure was partly created with BioRender.

## Results and Discussion

### Synthesis of yPEGs and yPEG-*b*-PLGA

In this work, we introduce the modification of PEGylated systems with ylides residues. For this purpose, we modified homopolymer HO-PEG-COOH and the frequently used amphiphilic block copolymer PLGA-*b*-PEG-COOH by the conjugation with a sulfur ylide containing tri- or pentapeptide. We opted for relatively small-sized sulfur ylides, which also exhibit significantly higher stability under physiological conditions than other ylide motifs we have investigated, including imino phosphoranes.^54^ Additionally, we accessed a PEG variant featuring a primary amide terminus to distinguish properties inherent to sulfur ylides from those arising specifically due to the terminal amide functionality present at the C-terminus of the peptide (Figure 2). To achieve this, the corresponding peptides displaying ylides residues were obtained by the reaction of *N*-α-Fmoc-l-aspartic acid α-*tert*-butyl ester (Fmoc-Asp­(OH)-OtBu) with a (cyanomethyl)-dimethylsulfonium bromide salt as previously reported in literature.^55^ The tri- 3Asp­(SY) and pentapeptide 5Asp­(SY) synthesis was carried out by solid phase peptide synthesis (SPPS). By using peptide-derived building blocks and solid-phase peptide synthesis to install the ylide tags, we anticipate a straightforward path to future good manufacturing practice (GMP) translation. Established protocols already exist for conducting peptide chemistry under GMP conditions, covering pharmacological-grade starting materials, validated purification and isolation methods (e.g., reverse-phase high-performance liquid chromatography (HPLC)), and standardized analytical assays. The overlapped signals of the methyl and methylene protons of the peptide were identified at around 2.8 ppm. The signals of the corresponding amide and protonated amine were observed in the low field revealing a correct integration (Figures S6 and 7). Prior to the conjugation of the sulfur ylide containing peptides to the polymers, we first synthesized the block copolymer PLGA-*b*-PEG-COOH utilizing the commercially available HO-PEG-COOH (*M*_w_ = 5 kDa) as macroinitiator for the Sn­(Oct)_2_ catalyzed ROP. A gel permeation chromatography (GPC) elugram revealed unimodal mass distribution giving a polymer with a low dispersity (*Đ*) and a molecular mass of *M*_n_ = 21.9 kDa. With the amphiphilic PLGA-*b*-PEG-COOH polymer in hand, the addition of the sulfur ylide containing tri- and pentapeptides was conducted by reacting the N-terminus of the peptide to the polymeric carboxylic α end-functionality prior to activation by means of *N*-hydroxysuccinimide (NHS) chemistry. The ^1^H NMR spectra revealed a signal corresponding to the methyl groups adjacent to the positively charged sulfonium around 2.80 ppm. To further demonstrate the sulfur ylide moiety presence in the polymeric chain, the vibrational band at 2173 cm^–1^ corresponding to the nitrile group (C≡N) could be observed (Figures S22 and 23). The successful incorporation of the peptide units to the polymeric chains was also confirmed by diffusion-ordered spectroscopy (DOSY) NMR. The proton signals of the dimethyl groups of the peptide at 2.80 ppm and the proton signals of the polymer revealed the same diffusion coefficient as the ones corresponding to the polymeric chain; signals of the free peptide were not observed, indicating the successful conjugation (Figure 2b­(i)-(ii)). The conjugation of both peptides to HO-PEG-COOH was achieved in a similar fashion as with the case of PLGA-*b*-PEG-COOH. The incorporation of the peptide in the polymeric PEG chain was confirmed by ^1^H NMR in which it observed the methyl signals of the sulfonium residue at 2.89–2.85 ppm (Figure 2c­(i)). Notably, in contrast to the unmodified HO-PEG-COOH, the peptide-PEG conjugates appeared UV-active upon GPC analysis of both conjugates by the appearance of the elugram compared to the nonactive HO-PEG-COOH precursor (Figure 2c­(ii)). The refractive index (RI) elugram also confirmed a shift to a higher molecular weight, while the dispersity of the polymer remained unchanged (Figure S15 and 16). It has been reported that small modifications of the end-functional group of PEGylated systems can lead to significant changes in the recognition of this polymer by anti-PEG Abs.^38^ These changes can be attributed to the modification of the hydrophilicity of the end-group of the polymeric chain. To distinguish properties inherent to the ylide residues from those arising specifically due to the terminal amide functionality, we accessed a PEG variant featuring a primary amide terminus. Besides serving as a sole reference, we speculated that the amide residue alone may represent a promising alternative to the commonly used methoxy terminus. This is due to the fact that amide groups display higher polarity compared to methoxy-terminated PEG, while remaining overall charge neutral. This characteristic is attractive for the design of nanocarriers for drug delivery, as it could avoid binding to charged biomacromolecules and the formation of the so-called protein corona. Hence, we also report the synthesis of two additional polymeric systems consisting of the chemical modification of the carboxylic acid to an amide group of HO-PEG-COOH to give HO-PEG-CONH_2_ and the subsequent ROP of l-lactide and glycolide to give PLGA-*b*-PEG-CONH_2_. For this purpose, HO-PEG-COOH was treated with isobutyl chloroformate and 4-methylmorpholine, allowing the in situ formation of an acid anhydride, and subsequently reacted with ammonium hydroxide to give a polymer with an amide as an end-functional group. Successful conversion to the primary amide on the PEG terminus was confirmed by Fourier transform infrared spectroscopy (FT-IR), in which the vibrational band at 1730 cm^–1^ corresponding to the C=O stretch of the carboxylic acid is not distinguished after the end-group modification (Figure 2d­(i)). By GPC analysis, a significant change in dispersity or molecular weight of the polymer was not observed due to degradation under the reaction conditions (Figure S19). After the successful amidation of HO-PEG-COOH, the amphiphilic block copolymer PLGA-*b*-PEG-CONH_2_ was synthesized in a fashion similar to that of PLGA-*b*-PEG-COOH by ROP, obtaining a polymer with an *M*_w_ of 19.6 kDa (Figure 2d­(ii)). The properties of the different polymers synthesized or modified are summarized in Table S1.

**Figure 2:**
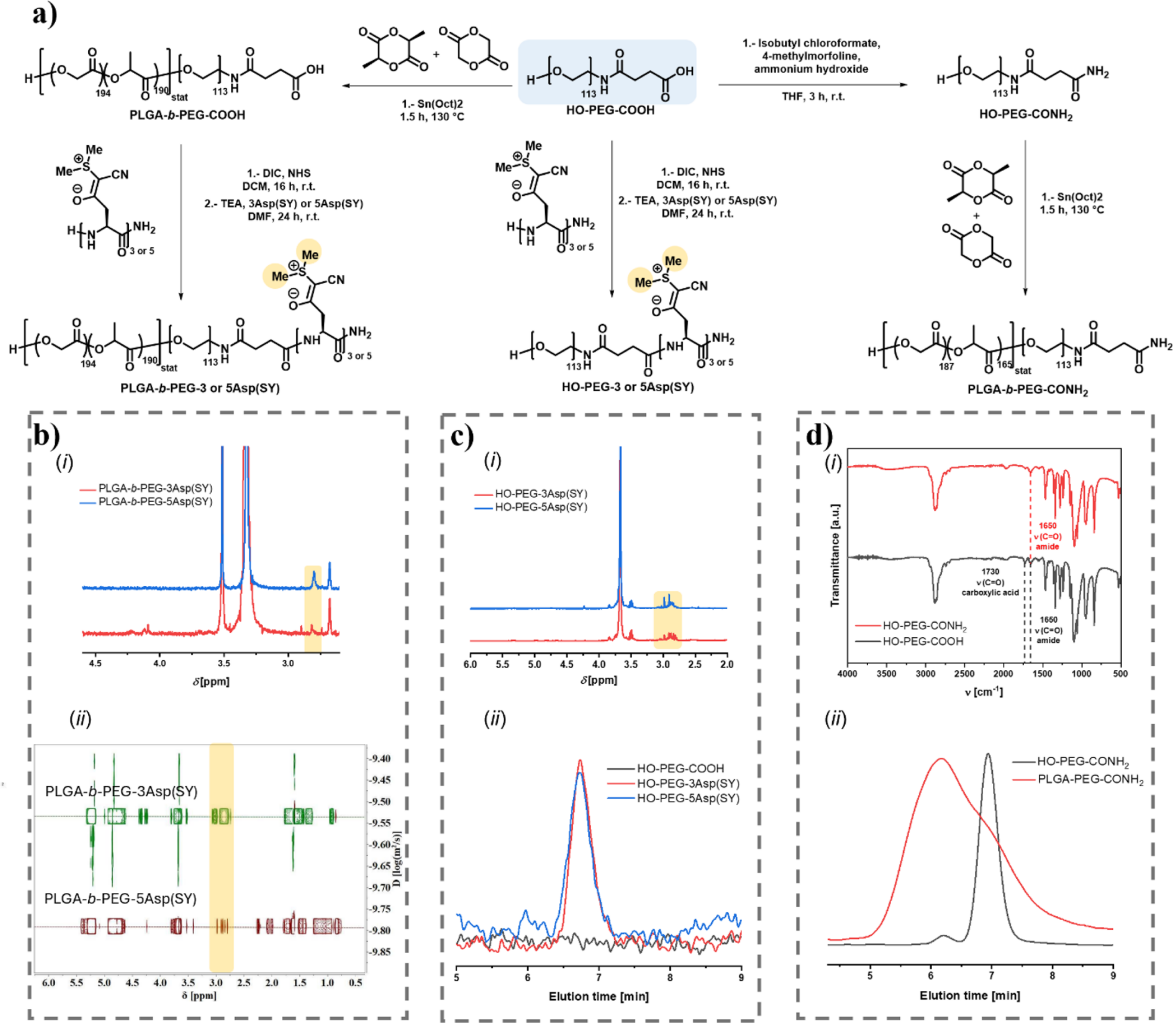
(a) Synthetic route for the conjugation of HO-PEG-COOH or PLGA-*b*-PEG-COOH with sulfur ylide-containing peptides and amidation of HO-PEG-COOH and subsequent ROP of l-lactide and glycolide. Characterization of the amide- and sulfur ylide-containing peptide polymers. (b) (i) Overlap ^1^H NMR spectra of PLGA-*b*-PEG-3Asp­(SY) and PLGA-*b*-PEG-5Asp­(SY); (ii) overlap DOSY spectra of PLGA-*b*-PEG-3Asp­(SY) and PLGA-*b*-PEG-5Asp­(SY). (c) (i) ^1^H NMR spectra of HO-PEG-3Asp­(SY) and HO-PEG-5Asp­(SY); (ii) UV–vis GPC elugrams of HO-PEG-COOH, HO-PEG-3Asp­(SY), and HO-PEG-5Asp­(SY). (d) (i) Overlay of FT-IR spectra of HO-PEG-CONH_2_ and HO-PEG-COOH before and after chemical modification of the carboxylic acid to amide; (ii) RI GPC elugrams of the macroinitiator HO-PEG-CONH_2_ and PLGA-*b*-PEG-CONH_2_.

### Nanoparticle Preparation and Characterization

In our study, we aimed to elucidate the effect of ylides on the characteristics of well-established PLGA-*b*-PEG nanocarriers. In order to compare the characteristics of the modified amphiphilic systems, PLGA-*b*-PEG-CONH_2_, PLGA-*b*-PEG-3Asp­(SY), and PLGA-*b*-PEG-5Asp­(SY) nanoparticles were constructed by the nanoprecipitation method using THF as a water-miscible solvent. DLS analysis revealed unimodal distributions of the hydrodynamic diameter of PLGA-*b*-PEG-CONH_2_, PLGA-*b*-PEG-3Asp­(SY), and PLGA-*b*-PEG-5Asp­(SY) nanoarticles with values of 195.4, 188.8, and 210.5 nm, respectively (Figure 3b). The zeta potential in Milli-Q water showed values of −12.60, −7.20, and −7.06 mV, respectively (Figure 3c). It is important to highlight that the most negative value was observed for the PLGA-*b*-PEG-CONH_2_ nanoparticles. However, this value is still not as negative as compared to other PLGA-*b*-PEG systems with the same molecular weight and mass fraction of the hydrophilic block with a different end-functional group.^22,56,57^ Interestingly, slightly more positive zeta potential values were observed for the nanoparticles made of block copolymers containing either 3Asp­(SY) or 5Asp­(SY) as end-functional groups. These results showed an enhancement in the stabilization of the electrostatic interactions in the shell of the particle by the overall neutrally charged sulfur ylide moieties. The physicochemical characterization of the nanoparticles is summarized in Table 1.

**Table 1:** Physicochemical Characterization of Nanoparticles Corresponding to PLGA-*b*-PEG-CONH_2_, PLGA-*b*-PEG-3Asp­(SY), and PLGA-*b*-PEG-5Asp­(SY)

			**ZP [mV]**			
**System**	***D***_**H**_ **[nm]**	**PDI**	pH 7.0^a^	pH 6.9^b^	pH 6.0^b^	pH 5.5^b^
PLGA-*b*-PEG-CONH_2_	195.4 ± 2.6	0.1	–12.6 ± 0.1	–1.2 ± 0.6	1.8 ± 0.8	–0.9 ± 0.4
PLGA-*b*-PEG-3Asp(SY)	188.8 ± 6.6	0.2	–7.2 ± 0.1	–0.5 ± 0.1	–0.7 ± 0.2	–1.1 ± 0.3
PLGA-*b*-PEG-3Asp(SY)	210.5 ± 1.1	0.2	–7.0 ± 0.2	–2.0 ± 0.4	–1.4 ± 0.4	–1.2 ± 0.4

a Milli-Q water.

b PBS buffer.

**Figure 3:**
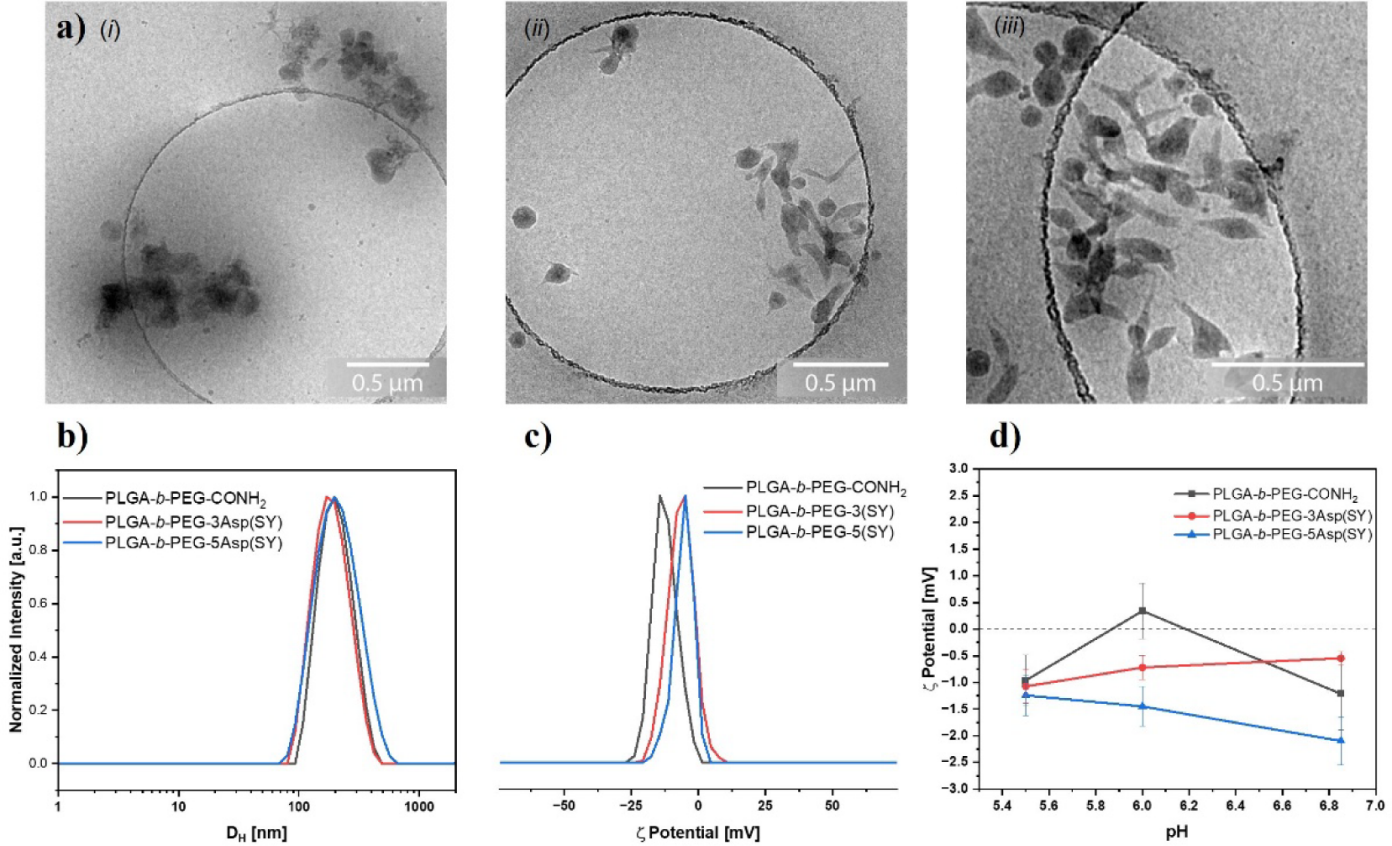
Characterization of the nanoparticles with amide- or sulfur ylide-containing peptides as end functional groups. (a) Cryo-TEM images of (i) PLGA-*b*-PEG-CONH_2_ NPs, (ii) PLGA-*b*-PEG-3Asp­(SY) NPs, and (iii) PLGA-*b*-PEG-5Asp­(SY) NPs. (b) Hydrodynamic diameter of PLGA-*b*-PEG-CONH_2_, PLGA-*b*-PEG-3Asp­(SY), and PLGA-*b*-PEG-5Asp­(SY) NPs in water. (c) Zeta potential values of PLGA-*b*-PEG-CONH_2_, PLGA-*b*-PEG-3Asp­(SY), and PLGA-*b*-PEG-5Asp­(SY) NPs in water. (d) Zeta potential values at different pH values of PLGA-*b*-PEG-CONH_2_, PLGA-*b*-PEG-3Asp­(SY), and PLGA-*b*-PEG-5Asp­(SY) NPs in PBS buffer solution.

The morphology of the nanoparticles was further analyzed by means of cryo-transmission electron microscopy (Cryo-TEM), in which more spherical nanoparticles were observed for the PLGA-*b*-PEG-CONH_2_ compared to the sulfur ylide systems, where a teardrop-shaped morphology was observed. This effect could be attributed to the reduced interfacial tension exhibited by the sulfur ylide in water (Figure 3a­(i-iii)). Another important characteristic is that the sulfur-ylide-modified polymeric nanoparticles were less likely to cluster together in the cryo-TEM images. Given previous research demonstrating that ylides enhance antifouling properties,^58^ it is plausible that the sulfur ylides in this context enhance particle dispersion by reducing interparticle adhesion, in contrast to amide-functionalized polymers. This phenomenon was also evident during sample dispersion following centrifugation, where nanoparticles containing sulfur ylide exhibited more rapid dispersion. Contrary to these results, the amide-functionalized polymers exhibit a propensity to aggregate postformation; although they presented the most negative zeta potential value, higher repulsion interactions between these nanoparticles would be expected. This aggregation could likely be caused by the relatively higher hydrophobicity of the amide group in the corona of the polymeric nanoparticle compared to the PLGA-*b*-PEG-3Asp­(SY) and PLGA-*b*-PEG-5Asp­(SY) systems, as well as the hydrogen-donor capabilities of the primary amide functionality. However, the aggregation of the amide nanoparticles is not a concern, as DLS results (Figure 3b) showed a monodisperse distribution after dispersion, confirming that the initial aggregation is reversible and does not impact the final stability. The change of the zeta potential of nanocarriers before approaching the targeted site directly impacts relevant properties including cellular uptake, stability, and protein adhesion.

One aspect that requires attention is the induced change in zeta potential caused by differences in pH levels, especially in cancer tissue, where the physiological pH value decreases compared to other sites, or due to interactions with biomacromolecules that form the so-called protein corona.

Therefore, it is important to address this problem by creating efficient nanocarriers that maintain their stability when they undergo these pH variations. We demonstrated that nanoparticles with a sulfur ylide peptide shell do not suffer from significant differences when decreasing the pH from 6.85 to 5.50, maintaining a modestly negative low zeta potential value. In contrast, the amide-functionalized block copolymer experiences changes from negative to positive values (Figure 3d).

### Nanoparticle Stability and Antifouling Protein in Physiological Conditions

To investigate the stability of ylide-displaying PLGA-*b*-PEG nanoparticles and to determine the impact of ylides on their stealth properties against nonspecific protein adhesion, we investigated their interaction with negatively charged bovine serum albumin (BSA) and positively charged lysozyme. Specifically, we examined PLGA-*b*-PEG-CONH_2_, PLGA-*b*-PEG-3Asp­(SY), and PLGA-*b*-PEG-5Asp­(SY) nanoparticles using field flow fractionation multiangle light scattering (FFF-MALS) to detect and monitor potential changes in their radius of gyration (*R*_g_). Combined with the hydrodynamic radius (*R*_h_), these measurements provide the shape factor (*R*_g_/*R*_h_), a key parameter used to assess whether nanoparticles are loaded with cargo or have surface attachments.^59^ The shape factor (*R*_g_/*R*_h_) is a critical indicator of particle structure. When this parameter equals 1, it suggests that the nanoparticles are roughly spherical or slightly elongated,^60,61^ with the radius of gyration located on the particle’s surface, coinciding with the hydrodynamic radius. However, when the shape factor increases, it indicates that the radius of gyration has shifted outward, often due to the addition of mass on the particle’s surface or periphery. Conversely, a lower shape factor (*R*_g_/*R*_h_ < 1) reflects mass concentrated closer to the particle’s center.^59^ This metric has been widely used to infer whether nanoparticles are loaded with cargo or possess surface modifications, as changes in the *R*_g_/*R*_h_ ratio provide insights into the spatial distribution of mass within or on the nanoparticles.^60,62^

We first monitored the variation in shape factor for the three nanoparticle types over 23 h to assess their stability at 37 °C in Dulbecco’s Phosphate Buffered Saline (DPBS) buffer solution. The results showed no significant changes in the *R*_g_/*R*_h_

ratio in any of the samples (Figure 4a), indicating that PLGA-*b*-PEG-CONH_2_, PLGA-*b*-PEG-3Asp­(SY), and PLGA-*b*-PEG-5Asp­(SY) remained stable under physiological conditions. The absence of changes in the shape factor confirms that the nanoparticles do not degrade during this period.

**Figure 4:**
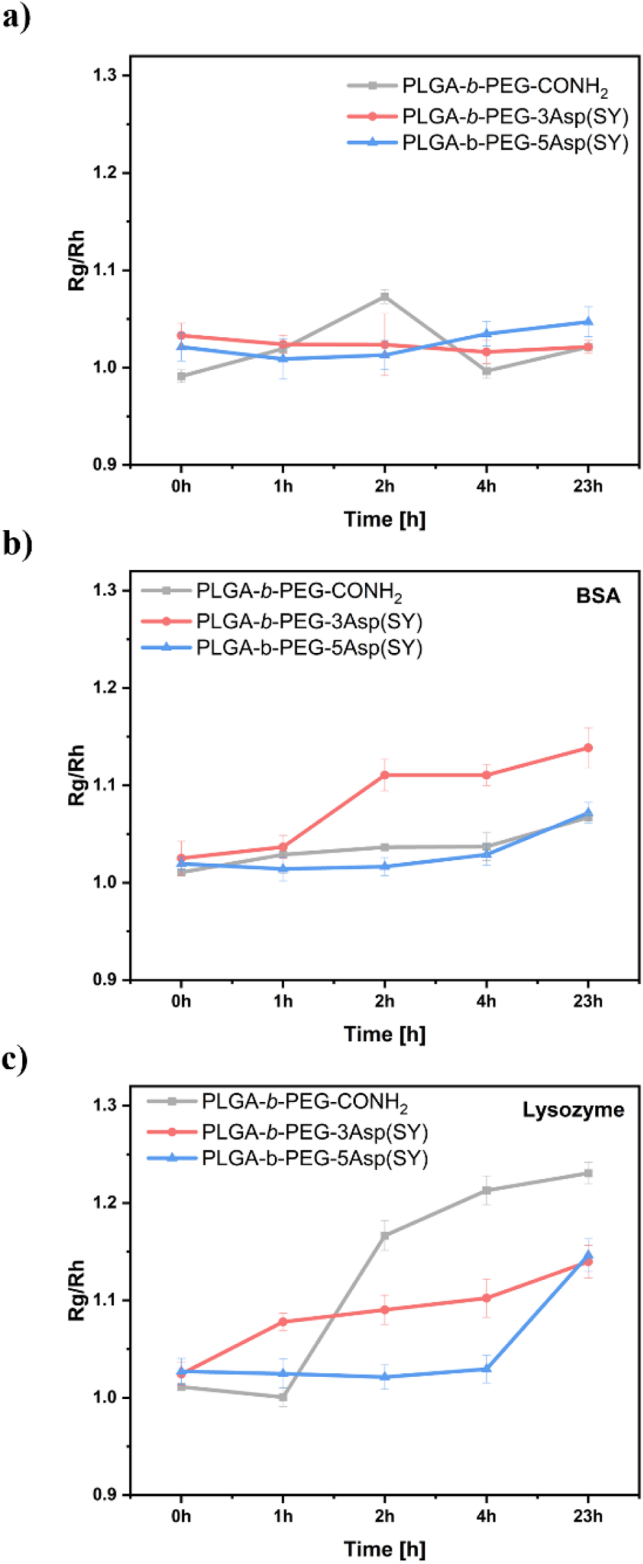
Shape factor (*R*_g_/*R*_h_) of PLGA-*b*-PEG-CONH_2_, PLGA-*b*-PEG-3Asp­(SY), and PLGA-*b*-PEG-5Asp­(SY) nanoparticles under (a) no treatment, b) BSA treatment, and (c) lysozyme treatment at time points 0, 1, 2, 4, and 23 h.

To assess the nanoparticle’s antifouling properties against BSA, the nanoparticles were incubated with this protein at a high concentration (*c* = 0.5 mg/mL) (Figure 4b). For PLGA-*b*-PEG-3Asp­(SY), a slight increase in shape factor was observed at time points 4 and 23 h, indicating possible BSA attachment to its surface. In contrast, no significant changes were detected for PLGA-*b*-PEG-CONH_2_ or PLGA-*b*-PEG-5Asp­(SY), suggesting minimal or no BSA binding. The repulsion between negatively charged BSA and functionalized nanoparticles likely explains the lack of attachment in these cases. The absence of detectable BSA binding to PLGA-*b*-PEG-CONH_2_ nanoparticles could be expected, as the zeta potential value was the most negative among all three systems, which could cause greater electrostatic repulsion. In the case of nanoparticles containing sulfur ylide, the antifouling property increases with the extension of the peptide sequence. The attachment of positively charged lysozyme was evaluated similarly (Figure 4c). After 2 h, increases in shape factor were noted for PLGA-*b*-PEG-CONH_2_, suggesting strong protein-nanoparticle interactions. The negatively charged nature of the nanoparticle could facilitate these interactions. In contrast, significant changes in *R*_g_/*R*_h_ value in the systems PLGA-*b*-PEG-3Asp­(SY) and PLGA-*b*-PEG-5Asp­(SY) are only observed after prolonged exposure to the protein (23 h). As in the case of the BSA experiment, a stronger antifouling property is observed for the nanoparticles containing sulfur ylide peptide termini. These results suggest that PLGA-*b*-PEG-3Asp­(SY) is suitable for short-term antifouling applications, whereas PLGA-*b*-PEG-5Asp­(SY) offers better long-term performance, as lysozyme attachment occurs only after extended incubation. Additionally, the stability of the nanoparticles was tested in cell culture medium containing Dulbecco’s Modified Eagle Medium-high glucose (DMEM) with 10% fetal bovine serum (FBS). Interestingly, the systems PLGA-*b*-PEG-3Asp­(SY) and PLGA-*b*-PEG-5Asp­(SY) showed high stability even after 24 h. Notably, the systems PLGA-*b*-PEG-3Asp­(SY) and PLGA-*b*-PEG-5Asp­(SY) showed high stability even after 24 h (Figures S40–42).

### Anti-PEG Antibody Binding

Finally, we determined the ability of terminally installed ylides to diminish or even prevent binding to anti-PEG Abs using two different isotypes of monoclonal antibodies: immunoglobulin M (IgM) and immunoglobulin G (IgG) with different specificities, namely, main-chain specific and terminal methoxy specific. In particular, we expected the latter to be greatly impacted by terminal ylide modification due to the enhanced hydrophilicity of ylide termini. To confirm the impact of terminal ylide residues on the inhibition of anti-PEG Abs recognition, we examined indirect ELISAs with the frequently used poly­(benzyl l-aspartate)-*block*-methoxy poly­(ethylene glycol) (PBLA-*b*-mPEG) (PEG *M*_w_ = 12 kDa, number of BLA units = 31) as a positive control. The four different block copolymers with varying PEG termini were precoated on ELISA plates (Maxisorp) for binding determination of anti-PEG Abs (IgM and IgG) with main-chain specific and terminal methoxy specific Abs. First, we investigated the binding of polymers to main-chain specific IgM (Figure 5a­(i)). Previously, we reported the immunogenicity of methoxy terminal PBLA-*b*-mPEG, namely the elicitation of anti-PEG IgM antibodies.^35^ Indeed, PBLA-*b*-mPEG exhibited highly immunogenic characteristics and high affinity to anti-PEG Abs. In contrast, main-chain specific anti-PEG IgM exhibited a lower tendency to bind to all three polymers: PLGA-*b*-PEG-CONH_2_, PLGA-*b*-PEG-3Asp­(SY), and PLGA-*b*-PEG-5Asp­(SY).

**Figure 5:**
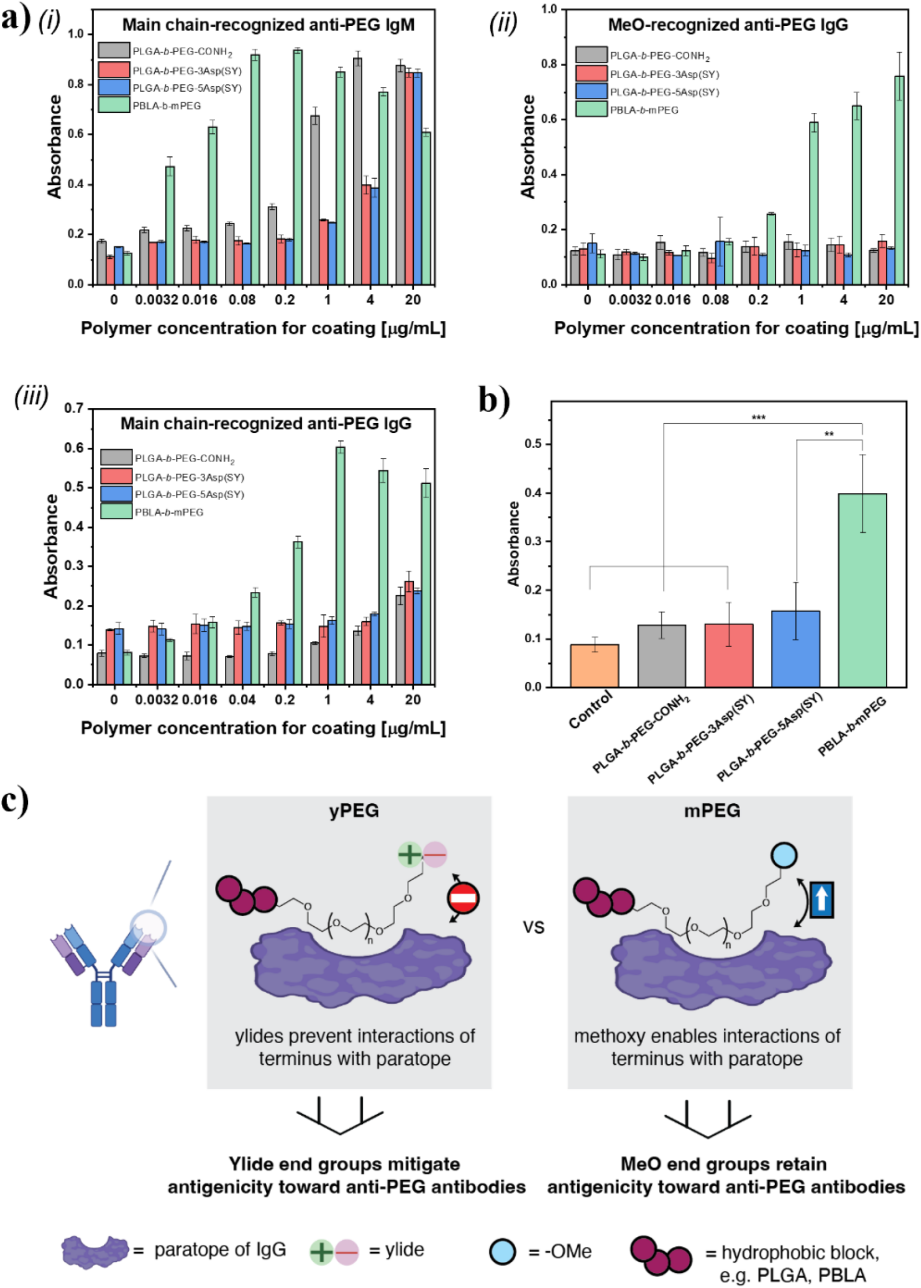
Bindings of a) (i) main-chain-specific anti-PEG IgM (3.0 μg/mL), (ii) terminal methoxy-specific anti-PEG IgG (3.0 μg/mL), and (iii) main-chain-specific anti-PEG IgG (3.0 μg/mL) to different terminal types of PEGs by indirect ELISA. Indicated polymer concentrations were used for plate coating at 4 °C overnight. Bound anti-PEG antibody was detected by either HRP-conjugated antimouse IgM or IgG (0.0125 μg/mL). (b) Each polymer was coated on the plate (20 μg/mL in EtOH/H_2_O). Sera were diluted 50 times (2.0 μL/100 μL in saline) for ELISA. *p* < 0.05. (c) Schematic illustration of the postulated mechanism how ylides, as termini of PEGs (yPEGs) as part of PEGylated systems, evade recognition by anti-PEG Abs. The figure was partly created with BioRender.

We attribute the lower antigenicity of yPEGs compared to mPEG to differences in recognition efficiency. PBLA-mPEG was most sensitive to anti-PEG IgM, likely due to its terminal methoxy group resembling PEG. In contrast, the polar ylide ends may exert slight repulsion toward main-chain-specific anti-PEG IgM, which prefers the nonpolar PEG terminus or backbone. Thus, strong binding to yPEGs likely requires a highly PEGylated surface. The primary amide-terminated block copolymer displayed binding at lower concentrations compared to the two ylide-displaying polymers, albeit significantly less than PBLA-*b*-mPEG. Next, we examined the binding of the block copolymers to MeO-specific IgG anti-PEG Abs (Figure 5a­(ii)). We observed no binding of methoxy-specific anti-PEG IgG; therefore, all three block copolymers fully inhibited methoxy-specific anti-PEG IgG recognition, not only emphasizing the utility of ylides as hydrophilic PEG termini but also highlighting the potential value of overall charge-neutral primary amides as terminal residues. In addition, comparison with PLGA-*b*-mPEG reveals the same trend (Figures S36 and S37). This additional control confirms that the observed effect is due to the terminal group, rather than the hydrophobic segment. Finally, we investigated the binding of yPEGs to main-chain-specific IgG antibodies (Figure 5a­(iii)). Main-chain-specific anti-PEG IgG also recognized all of the polymers; however, specific binding involves both recognition and physical attachment. While IgM molecules compensate for their lower antigen specificity through their high molecular weight, IgG antibodies rely on their inherently higher antigen specificity.^35^ We observed exactly this phenomenon, namely, that IgG shows less binding to yPEGs compared to main-chain-specific IgMs.

Next, we examined the binding of ylide-terminated systems to polyclonal anti-PEG IgM from mouse sera. For this purpose, sera were collected 7 days after immunization with PBLA-*b*-mPEG self-assemblies (dose of 0.1 mg/kg). Notably, polyclonal anti-PEG IgM elicited by PBLA-*b*-mPEG did not strongly recognize yPEGs, whereas polyclonal anti-PEG IgM recognized PBLA-*b*-mPEG (Figure 5b). Importantly, the antibodies were directed against mPEG rather than PEG, indicating that subtle structural modifications at the terminus can significantly alter PEG’s antigenicity.

The nature of the initial antigenic exposure plays a crucial role in shaping PEG recognition, rather than recognition being solely dependent on the PEG main chain itself. Placing a strongly hydrophilic yet overall charge-neutral residue at the terminus may affect the initial binding and recognition. Indeed, our results strongly indicate that introducing additional chemical complexity, such as modified end groups at the terminus, is an effective strategy for reducing PEG-specific recognition (Figure 5c). In the presence of anti-mPEG Abs, ylide-PEG is likely to be poorly captured or not captured at all, suggesting that tailored PEG modifications could help evade anti-mPEG Abs. We note that hydroxy-terminated PEG (HO-PEG) has been shown to reduce antigenicity; however, its terminal hydroxyl group may still trigger complement activation.^63,64^ While this study focused on the use of sulfur ylide-functionalized PEG as a strategy to mitigate PEG antigenicity, future work investigating the inhibition of recognition by anti-PEG antibodies in vivo would allow a more comprehensive understanding and further explore the promising translation to clinical tests. Previous studies have shown that the modification of PEGylated nanocarriers could combine the stealth effect of PEG with the inhibition of recognition by immune cells, which could lead to ineffective targeting.^65^

## Conclusions

This study presents ylide-functionalized PEG (yPEGs) as an innovative and scalable approach to mitigating PEG immunogenicity while maintaining stealth properties. By leveraging the unique chemical properties of ylides, including their strong hydrophilicity and charge neutrality, we introduce a highly effective yet straightforward strategy for reducing anti-PEG antibody recognition. Unlike other stealth polymer strategies that require extensive modifications to the polymer backbone, yPEGs retain PEG’s well-characterized properties while substantially enhancing immune evasion. We envision that yPEGs can be readily combined with recent strategies for PEG-backbone modifications^66^ such as the recent report on isomerization of PEG by the Frey group.^67^ With broad applicability in nanomedicine, biomaterials, and surface coatings, we foresee that yPEGs mark a significant advancement in PEGylation strategies, providing a next-generation solution for improving polymer biocompatibility.

## Supplementary Material



## Notes

Amino acid modification, peptide and polymer synthesis, polymer–peptide conjugation, and characterization information in the form of NMR spectra, GPC traces, FT-IR spectra, ESI-MS spectra, HRMS, fluorescence spectra, FFF-MALS chromatograms, indirect ELISA, and Cryo-TEM images (PDF)
