# Supplementary material for: Introducing Sulfur Ylides as Charge-Neutral Termini for Mitigating Poly(ethylene glycol) Antigenicity in Nanomedicine
Source: JACS Au. 2025 Sep 5;5(9):4378–88. doi: 10.1021/jacsau.5c00748 (PMC12457994; doi:10.1021/jacsau.5c00748)
Supplement: Supplementary file 1 [file au5c00748_si_001.pdf]

Supporting Information

Introducing Sulfur Ylides as Charge-Neutral Termini for Mitigating Poly(Ethylene Glycol) Antigenicity in Nanomedicine

Dulce M. Sánchez-Cerrillo,[a] Kouichi Shiraishi,*[b] Lucía Mallen-Huertas,[a] Remi Peters,[a] Daniela A. Wilson,*[a] and Kevin Neumann*[a]

[a] Institute for Molecules and Materials, Radboud University, The Netherlands.

[b] Research Center for Medical Sciences, the Jikei University School of Medicine, Japan

Table of Contents

Materials 3
Instrumentation 3
Synthesis 5
Characterization Data 15
Supplementary tables 38
References 40

Materials

All chemicals were used as received unless otherwise noted. Commercial α-hydroxy-ω-succinamic acid PEG varying molar masses PEG_{3k} and PEG_{5K} were purchased from Rapp Polymere GmbH and pre-dried over toluene before use. L-Lactide (Fluorochem EU, 95%) and Glycolide (Fluorochem EU, 99%) were recrystallized from toluene and dried in vacuo prior to usage. 7-Methyl-1,5,7,-triazabicyclo[4.4.0]dec-5-ene (mTBD, 95%), L-Fmoc-aspartic acid alpha-t-butyl ester (Fmoc-Asp(OH)-OtBu 97%), 2-bromoacetonitrile (97%), *O*-(Benzotriazol-1-yl)-*N,N,N',N'*-tetramethyluronium hexafluorophosphate (HBTU, 99%), *N*-Hydroxysuccinimide (NHS, 98%) were obtained from Fluorochem EU. Tin(II)-ethylhexanoate (Sn(Oct)2, 92.5-100%), dimethyl sulfide (99%), trifluoroacetic acid (TFA, 99%), acetic anhydride (99%), 4-methylmorpholine (99%), rink amide resin (100-200 mesh), deuterated chloroform ($CDCl_3$, 99.8% with 0.05% v/v TMS), and deuterated dimethyl sulfoxide (DMSO-d_6, 99.9% with 0.03% v/v TMS) were purchased from Sigma Aldrich. *N,N*-Diisopropylethylamine (DIPEA, 99.5%), *N,N*-dimethylformamide packaged under Argon (DMF, 99.8%), Isobutyl chloroformate (98%) were purchased from Thermo Fisher scientific. Pyperidine (99.5%), *N,N'*-Diisopropylcarbodiimide (DIC, 99%) were purchased from Biosolve BV. Propylphosphonic acid anhydride, 50% solution in ethyl acetate, was obtained from ABCR GmbH. Ammonium hydroxide was purchased from fisher scientific. Anhydrous solvents were obtained using a solvent purification system (SPS) from Actu-All Chemicals. The three different monoclonal anti-PEG Abs (AGP4, 6.3, and 15-2b) were purchased from the Institute of Biomedical Science at Academia Sinica (Taipei, Taiwan). Tris-buffer saline (TBS) was purchased from Sigma-Aldrich (Tokyo-Japan). Horse raddish peroxidase (HRP)-conjugated anti-mouse IgM and anti-mouse IgG were purchased from Tokyo Chemical Industry (TCI, Tokyo Japan).

Instrumentation

Nuclear Magnetic Resonance (NMR) spectroscopy was performed using a Bruker AVANCE III 400 MHz instrument, equipped with a BBFO probe. A known amount of material, approximately 5-10 mg were resuspended in 600 μL of DMSO-d_6 or $CDCl_3$ as NMR solvents. The reported chemical shifts are provided in ppm; residual ^1H resonance from deuterated solvent is used to reference the ^1H spectra with the methyl resonance of TMS at 0.0 ppm.

Gel permeation Chromatography (GPC) were recorded on a Shimadzu SEC, equipped with a guard column and a PL gel 5 µm mixed D column (Polymer Laboratories). Data was collected by a differential refractive index (RI) and UV (λ = 254 nm and λ = 280 nm) detection using *N,N*-dimethylacetamide (DMAc) at 50 °C as eluent, at 1.0 mL/min flow rate. The system was calibrated with PS standards.

Fourier transform infrared spectroscopy (FT-IR) spectra were measured with an spirit-T (Shimadzu) spectrophotometer equipped with a quest ATR diamond crystal. Measurements were performed in the transmittance mode and the data were analysed by LabSolutions IR 2.2 software. Spectra were scanned between 4000 and 500 cm^{-1}.

Electrospray ionization mass spectrometry (ESI-MS) was performed on a Single-Quad Thermo instrument equipped with a Thermo Scientific Accucore C18 (2.6 µm, 80 Å, 100 x 3 mm) column using 0.1 % formic acid in ACN and in Mili Q as eluents and differential refractive index (RI) and UV absorbance (λ = 254 nm). The mass spectrometer was operating in the positive ion mode.

Dynamic light scattering (DLS) Nanoparticles were characterized regarding hydrodynamic diameter (D_H), polydispersity index (PDI) and zeta potential (ZP) with the Zetasizer Nano ZS (Malvern Instruments) equipped with a 633 nm laser. Samples were measured by dynamic light scattering in (cuvettes) at a scattering angle of 173°. Samples were diluted 1:4 in miliQ water and equilibrated for 120 s at 25 °C before each measurement. Results were calculated using the refractive index (1.33) and viscosity (0.88 mPa s) of water. Three independent NP preparations with three runs per sample were measure and expressed as mean ± standard deviation.

Cryogenic electron microscopy (Cryo-TEM) was performed on a JEOL2100 with a single crystal filament LaB6 K-Type as electron source. The accelerating voltage was set to 200 kV and images were taken on the high magnification setting with a Gatan 833 Orius camera. Images were analyzed in ImageJ.

Fluorescence spectroscopy measurements were performed using a JASCO FP-8300ST Spectrofluorometer. The excitation wavelength was set at 339 nm. The slid width was 2.5 nm and spectra scan speed was set at 240 nm/min.

Field-Flow Fractionation Multi-Angle (FFF-MALS) The experiment was performed using Wyatt's Field Flow Fractionation – Multi Angle Light Scattering (FFF-MALS) system composed of a Shimadzu HPLC, Damn Heleos-II, Optilab T-rEX, and WyattEclipse AF4. The channel was linked to a Shimadzu LC-20A Prominence system with Shimadzu CTO20A injector. Wyatt DAWN HELEOS II light scattering detector (MALS) was set at various angles (12.9 °, 20.6 °, 29.6 °, 37.4 °, 44.8 °, 53.0 °, 61.1 °, 70.1 °, 80.1 °, 90.0 °, 99.9 °, 109.9 °, 120.1 °, 130.5 °, 149.1 °, and 157.8 °) UV light was set at wavelength 400 nm and the

membrane used was a regenerative cellulose 10 kDa obtained from Wyatt. The solvent employed was 20mM NaNO3 + 0.02% NaN3. QELS detector was installed at an angle of 140.1 ° and a Wyatt Optilab Rex refractive index detector. Prior measurements detectors were normalized using Bovine Serum Albumin.The software employed was ASTRA 6.1. FFF-MALS measurements were performed by injecting 20µl of sample and running a method with 1ml/min flow rate, 0.15ml/min inject flow rate, and crossflow 0.1 ml/min.

Microplate spectrophotometer Absorbance (450 nm) was recorded with a microplate reader (Multiskan GO, Thermo Scientific Inc. Ltd.).

Synthesis

(Cyanomethyl)-Dimethylsulfonium bromide (1)

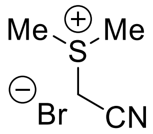

The synthesis of the sulfonium salt was done as previously reported with slight modifications.[1] Briefly, in a microwave vial 15 mL bromoacetonitrile was placed (6.0 g, 50.02 mmol, 1.00 eq.) and dimethyl sulfide (3.107 g, 50.02 mmol, 1.00 eq.). The mixture was stirred overnight. The obtained white solid was transferred to a falcon tube and resuspended three times in diethyl ether, subsequently centrifuged (4700 rpm, 4 °C, 10 min). Product was transferred to a glass vial, dried overnight under high vacuum. Yield: 8.27 g (91.86%).

^{1}H NMR [ppm] (400 MHz, DMSO-d$_6$): δ = 3.01 (s, 6H, CH_3), 4.79 (s, 2H, CH_2).

Fmoc-Asp(SY)-OtBu (2)

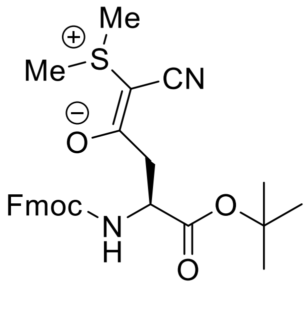

The synthesis of Fmoc-Asp(SY)-OtBu was synthesized as previously reported with slight modifications.[2] In a Schlenk flask previously dried overnight at 130 °C and cooled down at room temperature under argon flow Fmoc-Asp(OH)-OtBu was solubilized (9.415 g, 22.88 mmol, 1.0 eq.) in 99.89 mL of anhydrous

CH_2Cl_2. DIPEA (11.96 mL, 68.65 mmol, 3 eq.) were added dropwise to the solution followed by dropwise addition of T3P (≥50%w/w in EtOAc; 8.85 mL, 29.74 mmol, 1.30 eq.). The mixture was stirred fo 5 minutes before the addition of (cyanomethyl)dimethylsulfonium bromide (5.00 g, 27.46 mmol, 1.2 eq.). The suspension was stirred overnight at room temperature and then diluted with additional 100 mL of CH_2Cl_2. The organic layer was washed with 100 mL sat. aq. $NaHCO_3$ (1x), water (1x), and brine (1x), and subsequently dried over $NaSO_4$ and filtered. The crude was purified by flash column chromatography (CH_2Cl_2:MeOH 30:1) to give Fmoc-Asp(SY)-OtBu. Yield: 8.85 g (78.23%).

^{1}H NMR [ppm] (400 MHz, $CDCl_3$): δ = 7.30-7.81 (t, d, 8H, CH), 5.99 (d, 1H, NH), 4.59 (m, 1H, CH), 4.39-4.46 (b, 1H, CH), 4.21-4.35 (m, 2H, CH), 3.23 (d, 1H, CH_2), 2.94 (d, 1H, CH_2) 2.81-2.90 (d, 6H, CH_3), 1.50 (s, 9H, CH_3)

^{13}C NMR [ppm] (101 MHz, $CDCl_3$): δ = 189.00 (CO), 170.57 (CO), 119-156 (CH), 112 (C), 82.18 (C), 67.34 (CH_2), 51.30 (CH), 47.05 (CH), 40.53 (CH_2), 27.96 (CH_3).

ESI-MS: calculated for $C_{27}H_{30}N_2NaO_5S$ $[M+Na]^+$: 517.1768, found 517.16.

Fmoc-Asp(SY)-OH (3)

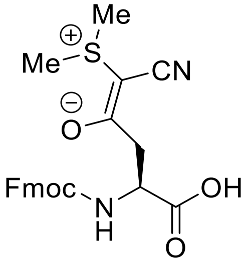

In a Schlenk flask it was solubilized Fmoc-Asp(SY)-OtBu (2) (3.0 g, 6.06 mmol, 1 eq.) in 7.54 mL of CH_2Cl_2 and mixture was cooled down to 0 °C. Trifluoroacetic acid (7.54 mL) were added to the mixture dropwise. The reaction was allowed to warm up to room temperature and stirred for 1 h. Product was precipitated in 50 mL of cold diethyl ether (-20 °C) and subsequently centrifuged at 4700 rpm, 4 °C, 10 min. Supernatant was disposed and pellet was transferred to a round-bottom flask with small addition of CH_2Cl_2. Excess of solvent was removed under reduced pressure. Product was dried overnight under high vacuum to give a white powder. Yield: 1.40 g (52.75 %).

^{1}H NMR [ppm] (400 MHz, $CDCl_3$): δ = 7.30-7.84 (t, d, 8H, CH), 6.01 (d, 1H, NH), 4.58 (m, 1H, CH), 4.42 (q, 2H, CH), 4.24 (t, 2H, CH), 3.41 (d, 1H, CH_2), 2.92 (d, 1H, CH_2), 2.89 (d, 6H, CH_3).

^{13}C NMR [ppm] (101 MHz, $CDCl_3$): δ = 189.31 (CO), 173.94 (CO), 119.73-156.54 (CH), 113.05 (C), 67.54 (C), 59.50 (CH), 50.38 (CH), 46.92 (CH), 40.25 (CH_2), 28.16 (CH_3)

ESI-MS: Calculated for $C_{23}H_{23}N_2O_5S^+$ $[M+H]^+$: 439.13, found 439.06.

Fmoc Solid Phase Peptide Synthesis (SPPS) of 3Asp(SY) (4) and 5Asp(SY) (5)

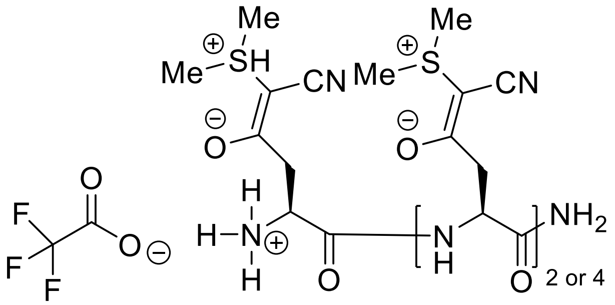

3Asp(SY) (**4**) and 5Asp(SY) (**5**) were synthesized by manual Fmoc-SPPS using HBTU activation procedure. Rink-amide resin was swollen and Fmoc deprotected using 20% piperidine in DMF (1 x 20 min). Afterwards the resin was washed with DMF (4 x 5 min). Fmoc-Asp(SY)-OH (**3**) was coupled by a preactivated solution containing 395 mg (3 eq.) of protected amino acid in 4 mL of DMF, 1.08 mL DIPEA 1 M solution in DMF (1.2 eq.), and 1.98 mL of HBTU 0.5 M solution in DMF (1.1 eq.). First amino acid was coupled overnight, successive AA couplings were performed during 2 hours. The resin was washed with DMF (3 x 5 min), and piperidine (20% in DMF) was added to the resin for Fmoc deprotection, and washed again with DMF (3 x 5 min) and (1 x 7 min). After last coupling and deprotection resin was washed with DMF (3 x 5 min), CH_2Cl_2 (3 x 5 min) and DMF (3 x 5 min). For peptide cleavage, the peptide was treated with TFA/CH_2Cl_2 (9:1) for 1 h. The resin was removed by filtration. The peptide was collected by precipitation in cold diethyl ether (-20 °C). Product was centrifuged at 4700 rpm, 4 °C, 10 min. Supernatant was discarded and peptide was resuspended in cold diethyl ether for a second centrifugation. Peptide was dried under high vacuum overnight.

For synthesis of 5Asp(SY) (**5**) and additional step was incorporated, remaining active amines after each coupling were capped with DMF/DIPEA/acetic acid anhydride (10:2:1, v:v:v).

3Asp(SY)

^{1}H NMR [ppm] (400 MHz, DMSO-d$_6$): δ = 8.59 (d, 1H, NH), 8.16 (d, 1H, NH), 8.08 (b, 3H, NH_3^+), 7.10 (s, 2H, NH_2), 4.56 (m, 2H, CH), 4.12 (m, 1H, CH), 2.75-2.88 (2d, 24H, CH_3, CH_2)

FT-IR [cm^{-1}]: 1557 (ν (NH)), 1660 (ν (C=O)), 2173 (ν (C≡N), 2935 (ν (CH)), 3018 (ν (CH)), 3289 (ν (NH))

HRMS (ESI): Calculated for $C_{24}H_{33}N_7O_6S_3H^+$ $[M+H]^+$ 612.1727, found 612.1727.

5Asp(SY)

^{1}**H NMR** [ppm] (400 MHz, DMSO-d$_6$): δ = 8.57 (t, 2H, NH), 8.15 (t, 2H, NH), 8.06 (b, 3H, NH_3^+), 6.86-7.14 (b, 2H, NH_2), 4.56 (b, 4H, CH), 4.12 (b, 1H, CH), 2.74-2.85 (2d, 40H, CH_2, CH_3)

FT-IR [cm^{-1}]: 1564 (ν (NH)), 1664 (ν (C=O)), 2167 (ν (C≡N), 2931 (ν (CH)), 3017 (ν (CH)), 3275 (ν (NH))

HRMS (ESI): Calculated for $C_{40}H_{53}N_{11}O_{10}S_5H^+$ [M+H]$^+$ 1008.2653, found 1008.2618.

Synthesis of PEG-3Asp(SY) (7) and PEG-5Asp(SY) (8)

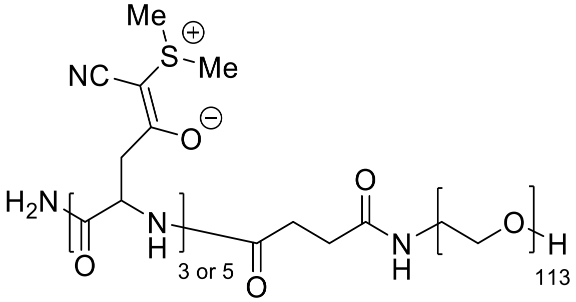

For activation of HO-PEG-COOH, HO-PEG-COOH (0.01 mmol, 50 mg) M_w = 5 kDa and NHS (0.05 mmol, 5.75 mg), were solubilized in 7.04 mL of anhydrous CH_2Cl_2. A stock solution containing 0.05 mmol of DIC in 0.1 mL of CH_2Cl_2 was added to the reaction mixture. The reaction proceeded for 16 h at room temperature under vigorous continuous stirring. Afterwards, the mixture was precipitated into 70 mL of cold diethyl ether (-20 °C) and centrifuged for 10 min at 4 °C and 4700 rpm. The supernatant was removed and the product was transferred into a glass vial to dry under high vacuum to yield HO-PEG-NHS (**6**).

For PEG-3Asp(SY) and PEG-5Asp(SY) coupling. In a microwave vial previously dried overnight at 130 °C and cooled down at room temperature under Argon stream, HO-PEG-NHS (0.003 mmol, 15 mg, 1.0 eq.) was solubilized in 1.45 mL of anhydrous DMF. 0.1 mL of a stock solution containing TEA in DMF (0.027 mmol, 9 eq.) were added into the reaction mixture and let stirred for 3 minutes. 3Asp(SY) or 5Asp(SY) (0.009 mmol, 3 eq.) were solubilized in 0.5 mL of anhydrous DMF and subsequently added into the reaction mixture. The reaction proceeded for 24 h at room temperature and the obtaining solution was precipitated twice into 20 mL of cold diethyl ether (-20 °C) and centrifuged for 10 min at 4 °C and 4700 rpm. Excess of solvent was removed under reduced pressure and product was dried overnight under high vacuum. Yield PEG-3Asp(SY): 7.71 mg (45.85 %), . Yield PEG-5Asp(SY): 8.45 mg (46.94 %).

PEG-3Asp(SY)

SEC: (DMAc, PS calibration). M_n = 9855 g mol^{-1}, Đ = 1.02

^{1}**H NMR** [ppm] (400 MHz, DMSO-d$_6$): 3.67 (s, 454H, CH_2), 2.89 (m, 18H, CH_3).

PEG-5Asp(SY)

SEC: (DMAc, PS calibration). M_n = 9999 g mol^{-1}, Đ = 1.02

^{1}H NMR [ppm] (400 MHz, DMSO-d_6): 3.67 (s, 454H, CH_2), 2.85 (m, 30H, CH_3).

Synthesis of PLGA-PEG-COOH (9) initiated by HO-PEG-COOH

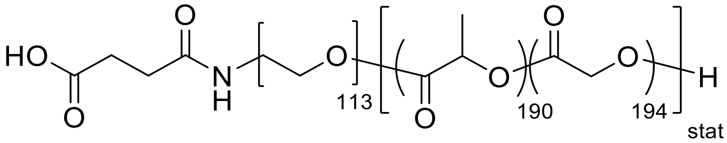

In a microwave vial previously dried overnight at 130 °C and cooled down to room temperature under Argon stream it was charged with L-lactide (300 mg, 2.08 mmol, 100 eq.), glycolide (241.60 mg, 2.08 mmol, 100 eq.) and HO-PEG-COOH (104.08 mg, 0.0208 mmol, 1 eq.). The vial was capped and evacuated and purged with vacuum and Argon three times, subsequently the mixture was placed in a metallic box at 130 °C. When the mixture was completely melted 0.1 mL of stock solution containing $SnOct_2$ (0.0208 mmol, 1 eq.) in 0.1 mL of dry toluene was added to the vial. After 1.5 h the mixture became very viscous, DMSO was added after cooling down to room temperature to solubilize the polymer. The polymer was dialyzed against deionized water for 2 days (membrane with a molecular weight cutoff (MWCO) = 3.5 kDa) and subsequently dried by lyophilization. Conversion L-lactide: 95%, conversion of glycolide: 97%.

SEC: (DMAc, PS calibration). M_n = 21,929 g mol^{-1}, Đ = 1.35.

^{1}H NMR [ppm] (400 MHz, $CDCl_3$): 5.23 (m, 190H, CH), 4.86 (br, 386H, CH_2), 3.67 (s, 454H, CH_2), 1.60 (d, 570, CH_3).

Synthesis of PLGA-PEG-3Asp(SY) (11) and PLGA-PEG-5Asp(SY) (12)

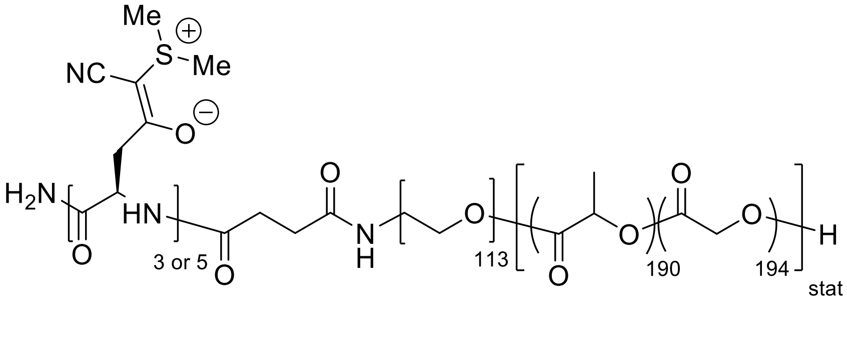

For activation of PLGA-PEG-COOH, in a Schlenk flask previously dried overnight at 130 °C and cooled down to room temperature under Argon stream PLGA-PEG-COOH (400 mg, 0.016 mmol, 1 eq.) and NHS (9.207 mg, 0.08 mmol, 5 eq.) were solubilized in 11.42 mL of anhydrous CH_2Cl_2. Afterwards, DIC (12.5

µL, 0.08 mmol, 5 eq.) were added into the reaction mixture. The reaction proceeded for 16 h at room temperature under vigorous continuous stirring. Afterwards, the product was precipitated twice into 90 mL of cold diethyl ether (-20 °C) and centrifuged for 10 min at 4 °C and 4700 rpm. The supernatant was removed and the product was transferred into a glass vial to dry under high vacuum to yield PLGA-PEG-NHS (**10**).

For the synthesis of PLGA-PEG-3Asp(SY) and PLGA-PEG-5Asp(SY) coupling, in a microwave vial previously dried overnight at 130 °C and cooled down to room temperature under Argon stream, PLGA-PEG-NHS (110 mg, 0.0044 mmol, 1 eq.) was solubilized in 10 mL of anhydrous DMF. TEA (5.5 µL, 0.0396 mmol, 9 eq.) were added into the reaction mixture and let stirred for 3 minutes. 3Asp(SY) or 5Asp(SY) (0.0132 mmol, 3 eq.) were solubilized in 5 mL of anhydrous DMF and subsequently added into the reaction mixture. The reaction proceeded for 24 h at room temperature. The polymer was purified by dialysis against deionized water for 2 days (membrane with a molecular weight cutoff (MWCO) = 3.5 kDa) and subsequently dried by lyophilization. Yield PLGA-*b*-PEG-3Asp(SY): 101.20 mg (75.31 %), . Yield PLGA-*b*-PEG-5Asp(SY): 101.44 mg (74.52 %).

PLGA-PEG-3Asp(SY)

SEC: (DMAc, PS calibration). M_n = 17,624 g mol^{-1}, Đ = 1.60.

^{1}H NMR [ppm] (400 MHz, DMSO-d$_6$): 5.23 (m, 190H, CH), 4.92 (br, 388H, CH_2), 3.52 (s, 454H, CH_2), 2.81 (s, 18H, CH_3) 1.49 (d, 570, CH_3).

PLGA-PEG-5Asp(SY)

SEC: (DMAc, PS calibration). M_n = 17,585 g mol^{-1}, Đ = 1.56.

^{1}H NMR [ppm] (400 MHz, DMSO-d$_6$): 5.23 (m, 190H, CH), 4.92 (br, 388H, CH_2), 3.52 (s, 454H, CH_2), 2.79 (s, 30H, CH_3) 1.49 (d, 570H, CH_3).

End group modification of HO-PEG-COOH to give HO-PEG-CONH$_2$ (13)

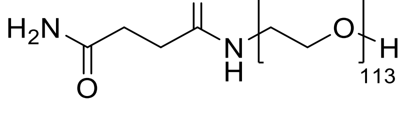

For the amidation of HO-PEG-COOH, in a Schlenk flask it was charged with HO-PEG-COOH M_w = 5kDa (200 mg, 0.04 mmol, 1 eq.) and purged and evacuated with vacuum and Argon three times. The polymer

was solubilized in 1 mL of anhydrous THF and let stirred for 10 minutes under Argon stream at 0 °C. Afterwards, isobutyl chloroformate (6.2 µL, 0.048 mmol, 1.2 eq.) and 4-methylmorfoline (5.27 µL, 0.048 mmol, 1.2 eq.) were added to the reaction mixture and let stir for one more hour at 0 °C. Subsequently, 1 mL of ammonium hydroxide was added and reaction proceeded for 3 hours at room temperature. The solution was concentrated under reduced pressure. Product was redissolved in small amount of CH_2Cl_2 and dried over $MgSO_4$, filtered and concentrated under reduced pressure. Modified PEG was dried by resuspension in toluene and dried under reduced pressure for removal of the azeotropic mixture of water and toluene. The polymer was dried under high vacuum overnight to yield HO-PEG-$CONH_2$. Yield: 115.0 mg (57.5 %).

SEC: (DMAc, PS calibration). $M_n = 9{,}329$ g mol^{-1}, Đ = 1.009.

^{1}H NMR [ppm] (400 MHz, $CDCl_3$): 3.66 (s, 454H, CH_2)

PLGA-PEG-$CONH_2$ (14) initiated by HO-PEG-$CONH_2$

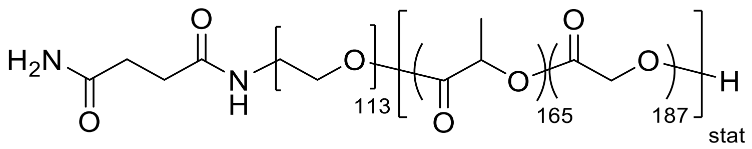

A microwave vial previously dried overnight at 130 °C it was cooled down to room temperature by continuous Argon stream. The vial was charged with HO-PEG-$CONH_2$ (50 mg, 0.01 mmol, 1 eq.), L-lactide (144.13 mg, 1 mmol, 100 eq.) and glycolide (116.07 mg, 1 mmol, 100 eq.). The vial was capped and evacuated and purged with vacuum and Argon three times, subsequently the mixture was placed in a metallic box at 130 °C. When the mixture was completely melted 0.1 mL of stock solution containing $SnOct_2$ (0.01 mmol, 1 eq.) in 0.1 mL of dry toluene was added to the vial. After 2 h the mixture became very viscous, DMSO was added after cooling down to room temperature to dilute the mixture. The polymer was purified by precipitated once in cold MeOH (-20 °C) and twice in cold diethyl ether (-20 °C) and centrifuged for 10 min at 4 °C and 4700 rpm. After last precipitation, product was transferred to a glass vial and dried under high vacuum overnight. Conversion L-lactide: 83%, conversion of glycolide: 94%.

SEC: (DMAc, PS calibration). $M_n = 12{,}143$ g mol^{-1}, Đ = 1.56.

^{1}H NMR [ppm] (400 MHz, $CDCl_3$): 5.21 (m, 165H, CH), 4.85 (br, 374H, CH_2), 3.67 (s, 454H, CH_2), 1.61 (d, 495, CH_3).

mPEG-PLGA initiated by mPEG-OH

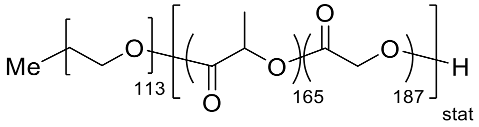

A microwave vial previously dried overnight at 130 °C it was cooled down to room temperature by continuous Argon stream. The vial was charged with HO-mPEG (50 mg, 0.01 mmol, 1 eq.), L-lactide (144.13 mg, 1 mmol, 100 eq.) and glycolide (116.07 mg, 1 mmol, 100 eq.). The vial was capped and evacuated and purged with vacuum and Argon three times, subsequently the mixture was placed in a metallic box at 130 °C. When the mixture was completely melted 0.1 mL of stock solution containing $SnOct_2$ (0.01 mmol, 1 eq.) in 0.1 mL of dry toluene was added to the vial. After 1.5 h the mixture became very viscous, DMSO was added after cooling down to room temperature to dilute the mixture and quenched with four-fold excess of benzoic acid. The polymer was purified by dialysis against deionized water for 2 days (membrane with a molecular weight cutoff (MWCO)= 3.5 kDa)) and subsequently dried by lyophilization. Conversion L-lactide: 74%, conversion of glycolide: 76%.

SEC: (DMAc, PS calibration). M_n = 12,197 g mol^{-1}, Đ = 1.41.

^{1}H NMR [ppm] (400 MHz, $CDCl_3$): 5.21 (m, 148H, CH), 4.85 (br, 304H, CH_2), 3.67 (s, 454H, CH_2), 1.61 (d, 447, CH_3).

Nanoparticle formulation

The nanoparticles were produced by nanoprecipitation method in a water-miscible solvent, using THF. An amount of 12 mg of the block copolymer were dissolved in 2 mL of THF to give a final concentration of 6 mg/mL. This mixture was added dropwise to a solution containing 1 mL of miliQ water and 1 mL of ethanol while sonicating (45kHz, 80 W), after addition the cloudy suspension continued in sonication for 3 additional minutes. The suspension was uncovered and magnetic stirring overnight to evaporate the organic solvent. Nanoparticles were washed three times by centrifugation and resuspension in miliQ water (10,000 rpm, 10 min). For DLS analysis, 0.3 mL of the NP suspension was diluted in 0.7 mL of miliQ water.

Protein fouling of PLGA-PEG-3Asp(SY) and PLGA-PEG-5Asp(SY) nanoparticles with BSA and lysozyme.

Protein fouling of PLGA-*b*-$CONH_2$, PLGA-*b*-PEG-3Asp(SY) and PLGA-*b*-PEG-5Asp(SY) was analyzed by FFF-MALS accordingly, concentrated suspension of nanoparticles were diluted in Dulbecco's phosphate-buffered saline (DPBS) solution (pH 7.2) to give a final concentration of 1 mg/mL. Subsequently, two solutions containing 3.6 mg of lysozyme in 3.6 mL of DPBS and 3.6 mg of BSA in 3.6

mL of DPBS were prepared to give a concentration of 1 mg/mL for each protein. The stock solutions of the nanoparticles and the proteins were mixed in a ratio of 1:1 (v:v) each to give a final concentration of the nanoparticles and proteins of 0.5 mg/mL. Samples were measured at the beginning of the experiment and subsequently incubated at 37 °C. Measurements were made at different time points placing the sample immediately in the incubator after each measurement.

CMC determination of PLGA-PEG-3Asp(SY) and PLGA-PEG-5Asp(SY)

Fluorescence spectroscopy was used to determine the critical micelle concentration of PLGA-PEG-3Asp(SY) and PLGA-PEG-5Asp(SY). A stock solution containing 1.21 mg of pyrene in 5 mL of THF was prepared to give an initial concentration of 0.0012 M. Subsequently, 0.1 mL of the pyrene solution was diluted in 100 mL of deionized water, THF was evaporated to give a final concentration of pyrene in water of 1.2×10^{-6} M. Solutions of polymer in water at 0.0125, 0.01, 0.0075, 0.005, and 0.0025 mg/mL were prepared. Polymers and pyrene solutions were mixed to give a final pyrene concentration of 6×10^{-7} M. After mixture samples were left to stand to equilibrate overnight at room temperature and subsequently measured in emission mode by monitoring the change of the pyrene emission spectra intensities at Ii (λ = 372 nm) and Iiii (λ= 382.6). Values obtained are $CONH_2$ higher than compared to the ones reported in literature, this could be due to the increase of hydrophilicity of the end-functional group requiring higher polymer concentrations towards the formation of micelle.[3]

CMC PLGA-*b*-PEG-3Asp(SY) 0.01 mg/mL

CMC PLGA-*b*-PEG-5Asp(SY) 0.0075 mg/Ml

Physiological stability of nanoparticles

Physiological stability of PLGA-*b*-$CONH_2$, PLGA-*b*-PEG-3Asp(SY) and PLGA-*b*-PEG-5Asp(SY) was analyzed by Cryo-TEM accordingly, concentrated suspension of nanoparticles were diluted in a solution of cell culture medium containing Dulbecco's modified eagle's medium-high glucose (DMEM) with 10% fetal bovine serum (FBS) to give a final concentration of the nanoparticles of 0.5 mg/mL in Eppendorf tubes (1.5 mg/mL). Subsequently, the Eppendorf tubes were incubated at 37 °C. After 2 and 24 hours the sample was centrifuged and washed 2 times for the removal of the cell culture medium. Cryo-TEM samples were prepared and photographed afterwards.

Indirect ELISA procedure

We used three different monoclonal anti-PEG Abs (AGP4, 6.3, and 15-2b). AGP4 is mouse IgM antibody, and 6.3 and 15-2b are mouse IgG antibodies. AGP4 and 6.3 antibodies are main-chain-specific antibodies, and 15-2b antibody are terminal-methoxy-specific antibody. All procedures for ELISA were examined in triplicates and performed at 24–25°C unless otherwise noted. To prepare PEG-immobilized 96-well plates, we dissolved PLGA-*b*-PEG-3Asp(SY), PLGA-*b*-PEG-5Asp(SY), and PLGA-*b*-PEG-$CONH_2$ in THF. And the THF soluion was diluted with H_2O/EtOH (1/3 = v/v) as 1.0 mg/mL of a stock solution. The stock solution was further diluted with H_2O/EtOH (1/1 = v/v) to prepare 1 – 100 μgmL polymer solution. The polymer solution was added to 96 plate-wells (e.g., 100 μL of 20 μg/mL solution) and kept at 4°C for overnight. The wells were washed three times with the wash solution (tris-buffer saline (TBS), pH = 8.0), and were blocked with 1% BSA (in 50 mM TBS, pH = 8.0) for 1 h. After undergoing three washings, the wells were filled with freshly prepared either a solution of monoclonal anti-PEG Abs in Dulbecco's Phosphate Buffered Saline (D-PBS) or a 50 times diluted sera which contained mPEG-PBLA-induced anti-PEG IgM (stocked sera). After 1h, we washed the wells three times, and we added to a solution of horseradish-peroxidase (HRP)-conjugated anti-mouse IgM antibody (0.0125 μg/mL) or a solution of HRP-conjugated mouse IgG antibody (0.0125 μg/mL). After 1h, we washed the wells three times with the washing solution. To the wells, we added a solution of 3, 3', 5, 5'-tetramethylbenzidine (TMB) (100 μL). After 15 min, we added 0.36 N H_2SO_4 (100 μL) to the wells in order to stop the reaction.

Characterization Data

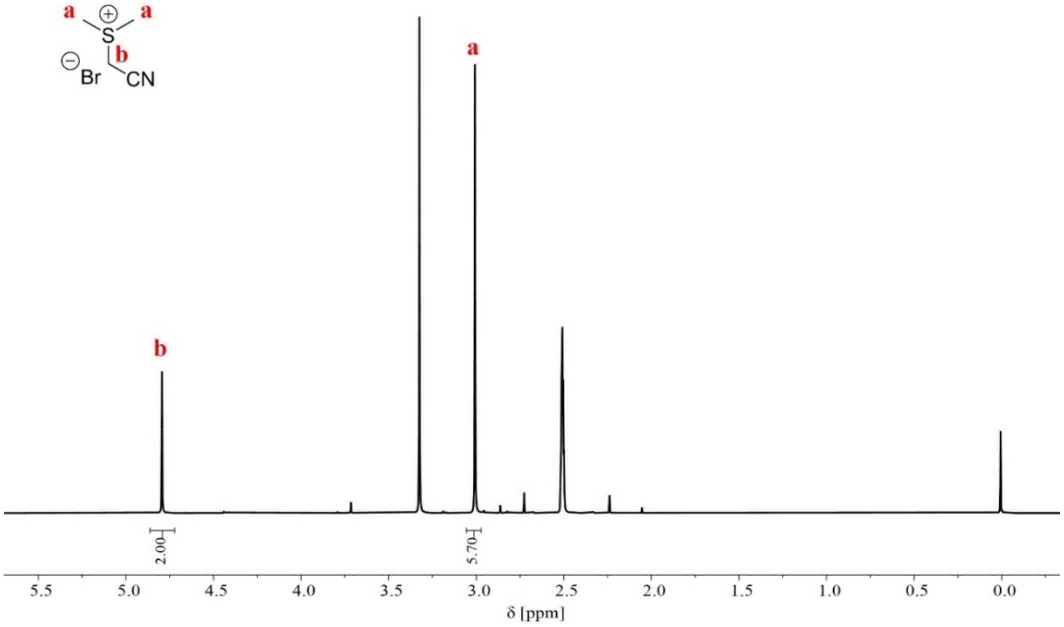

Figure S1: ^{1}H NMR spectrum (400 MHz, DMSO-d_6) of (Cyanomethyl)-Dimethylsulfonium bromide.

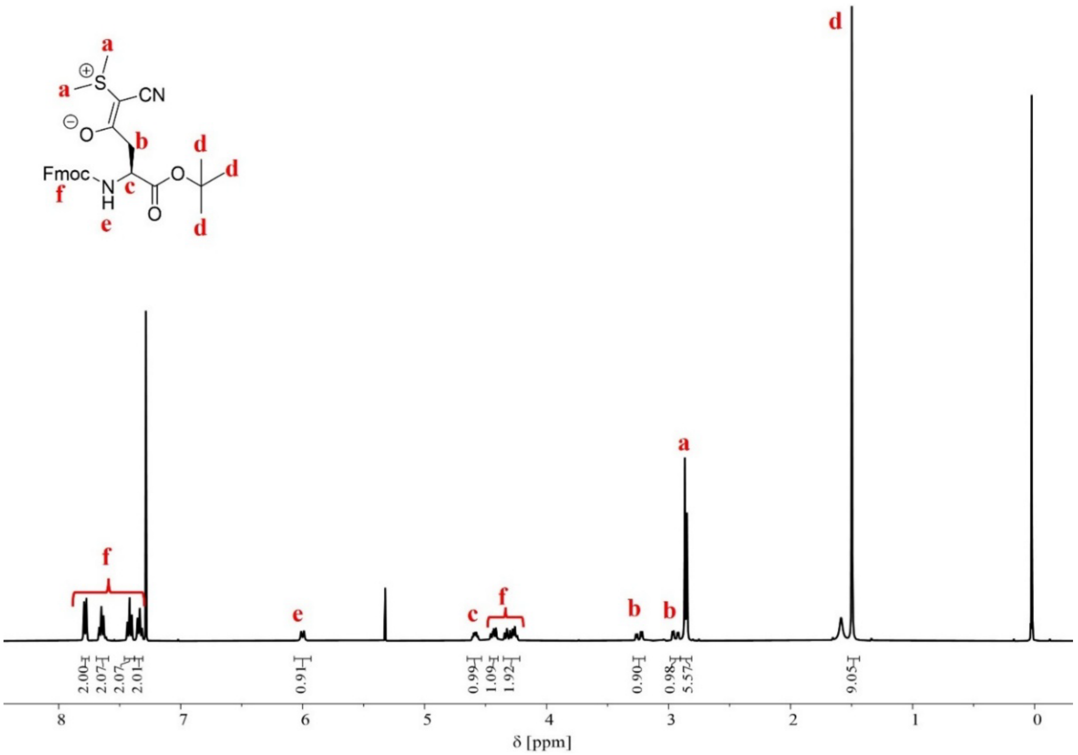

Figure S2: ^{1}H NMR spectrum (400 MHz, $CDCl_3$) of Fmoc-Asp(SY)-OtBu.

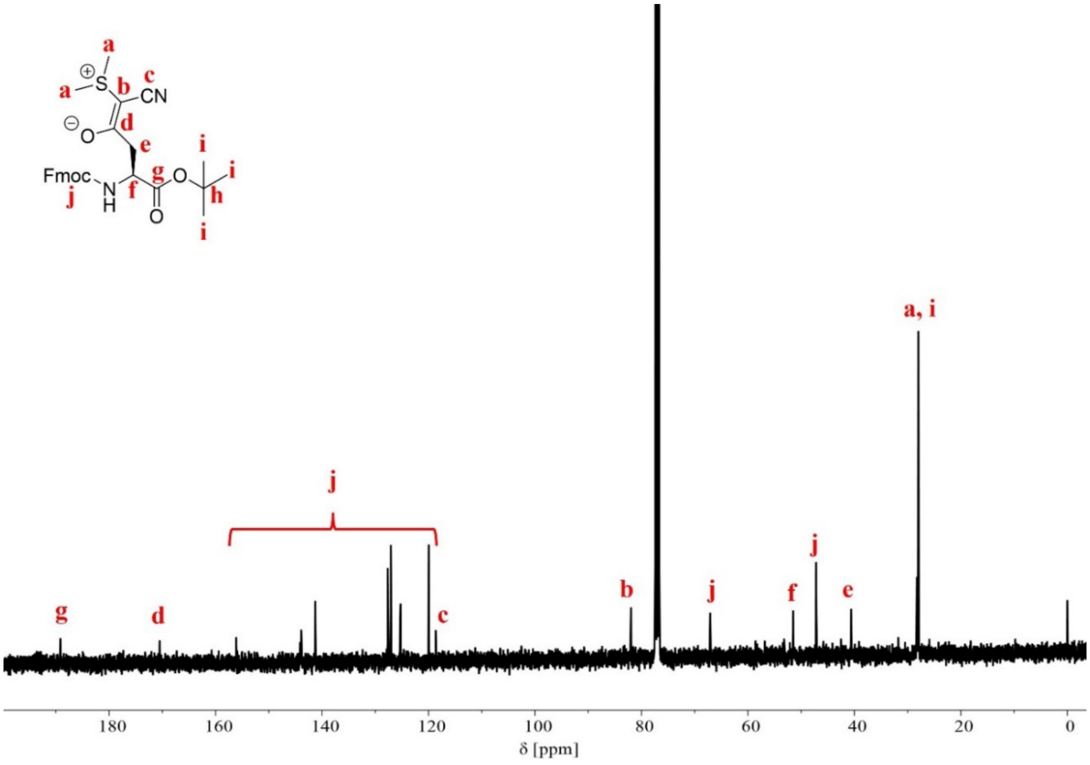

Figure S3: ^{13}C NMR spectrum (101 MHz, $CDCl_3$) of Fmoc-Asp(SY)-OtBu.

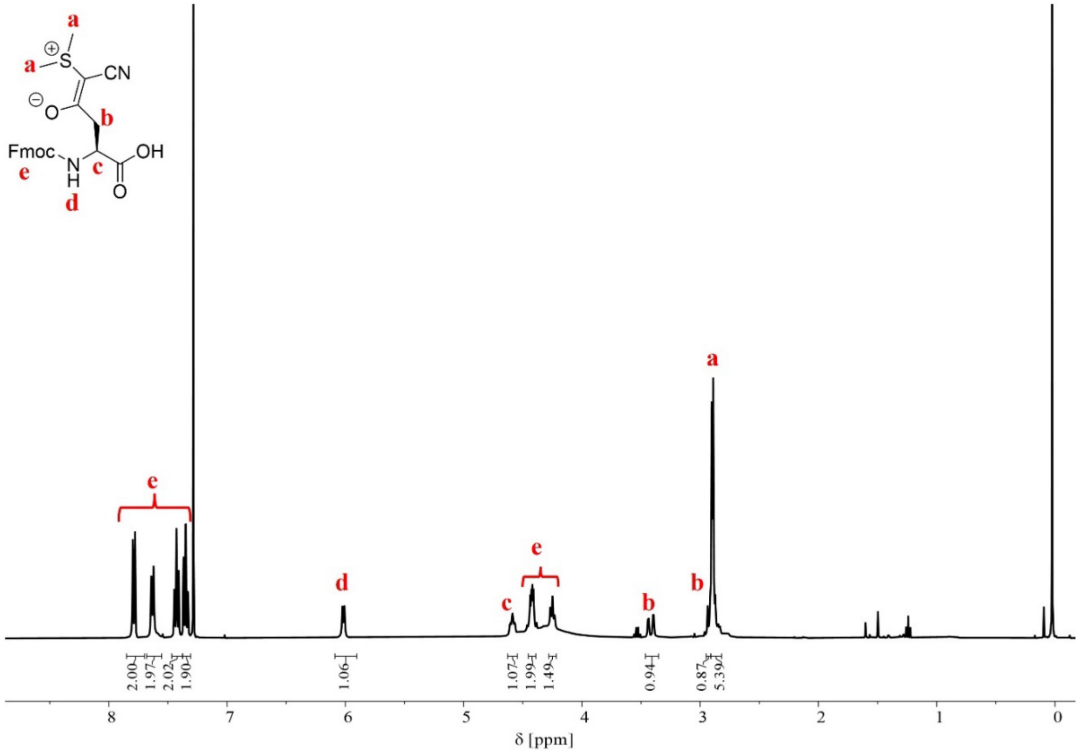

Figure S4: ^{1}H NMR spectrum (400 MHz, $CDCl_3$) of Fmoc-Asp(SY)-OH.

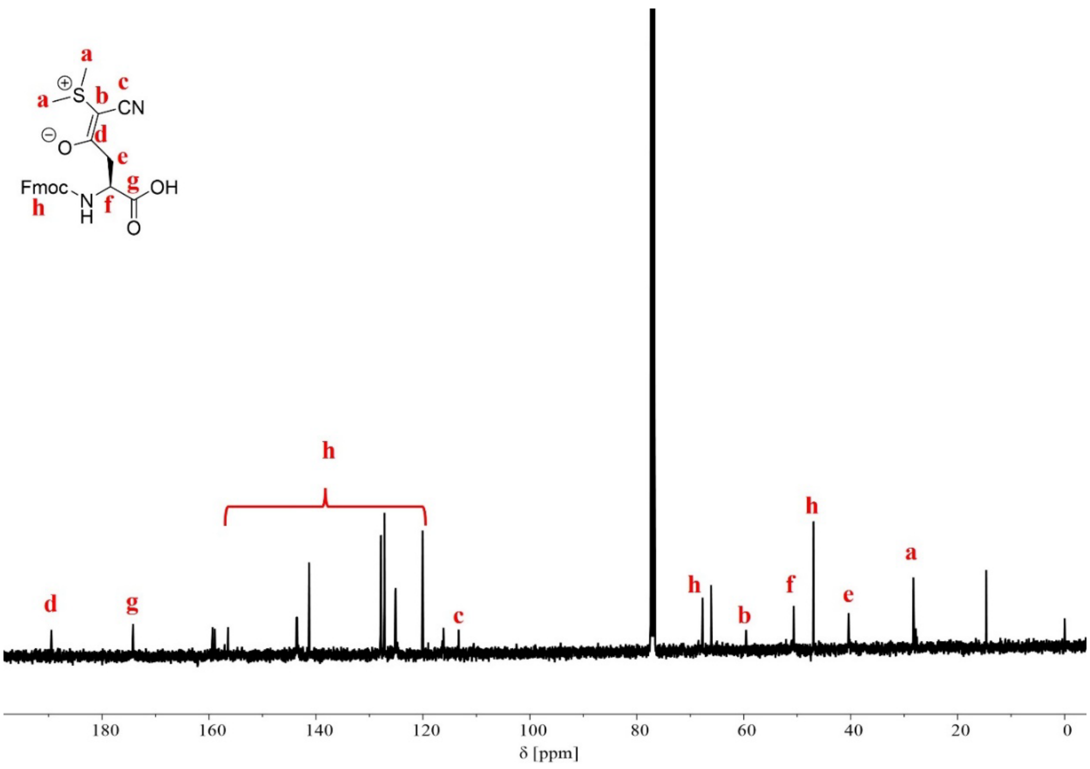

Figure S5: ^{13}C NMR spectrum (101 MHz, $CDCl_3$) of Fmoc-Asp(SY)-OH

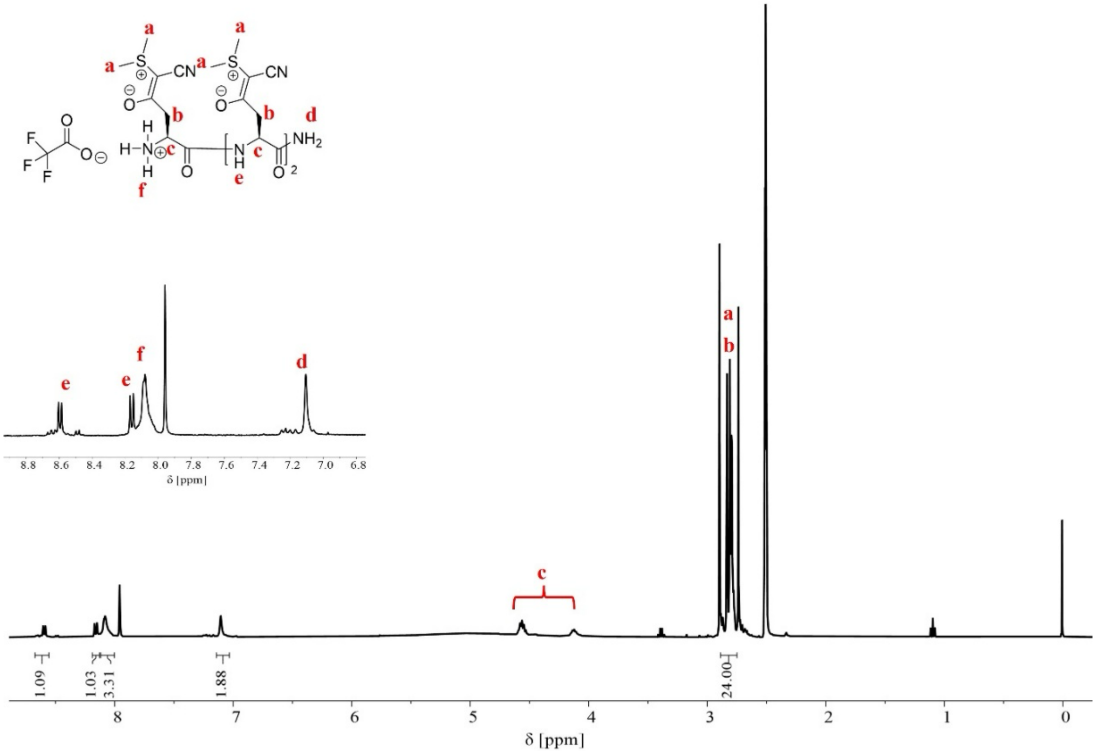

Figure S6: ^{1}H NMR spectrum (400 MHz, DMSO-d_6) of 3Asp(SY).

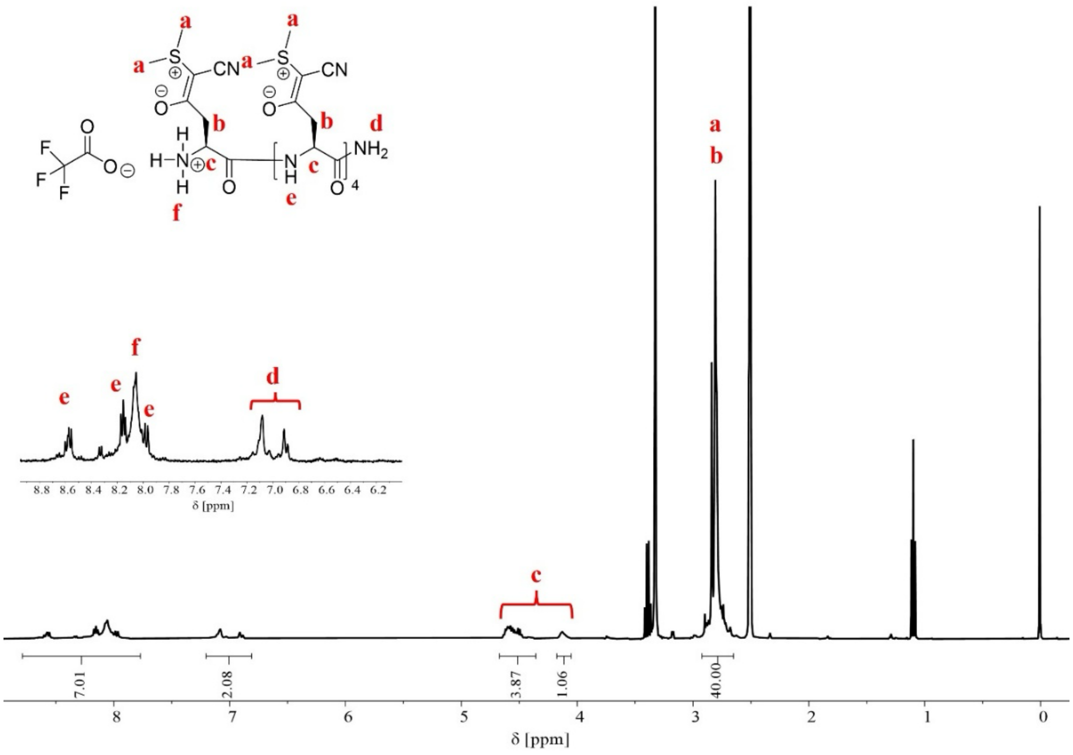

Figure S7: ^{1}H NMR spectrum (400 MHz, DMSO-d_6) of 5Asp(SY).

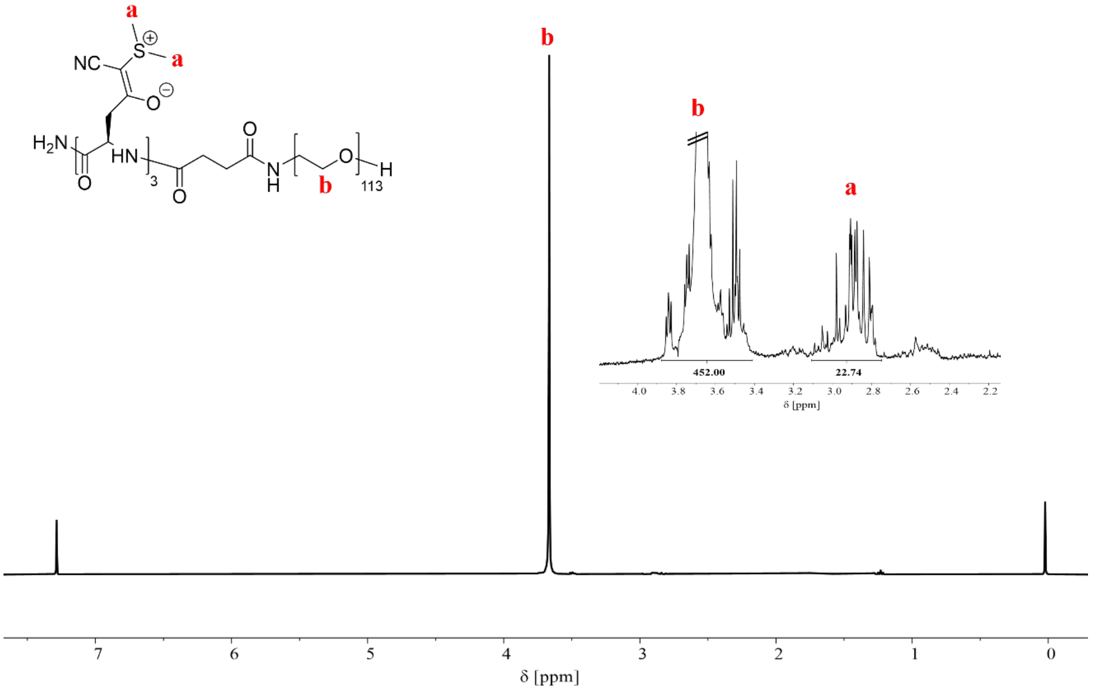

Figure S8: ^{1}H NMR spectrum (400 MHz, $CDCl_3$) of PEG-3Asp(SY).

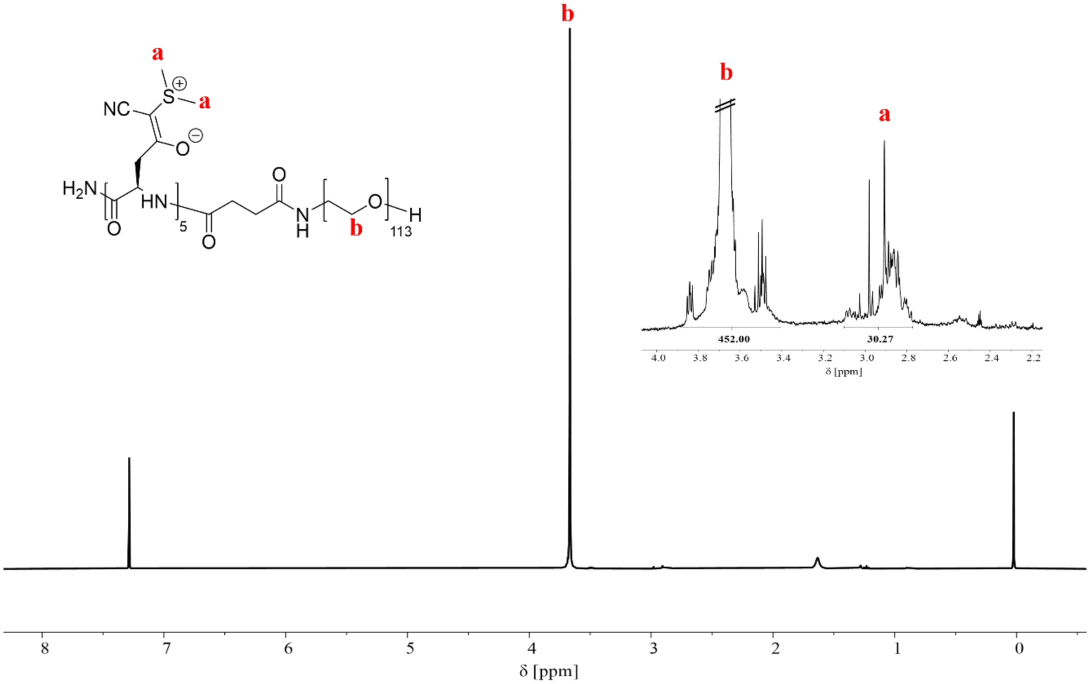

Figure S9: ^{1}H NMR spectrum (400 MHz, $CDCl_3$) of PEG-5Asp(SY).

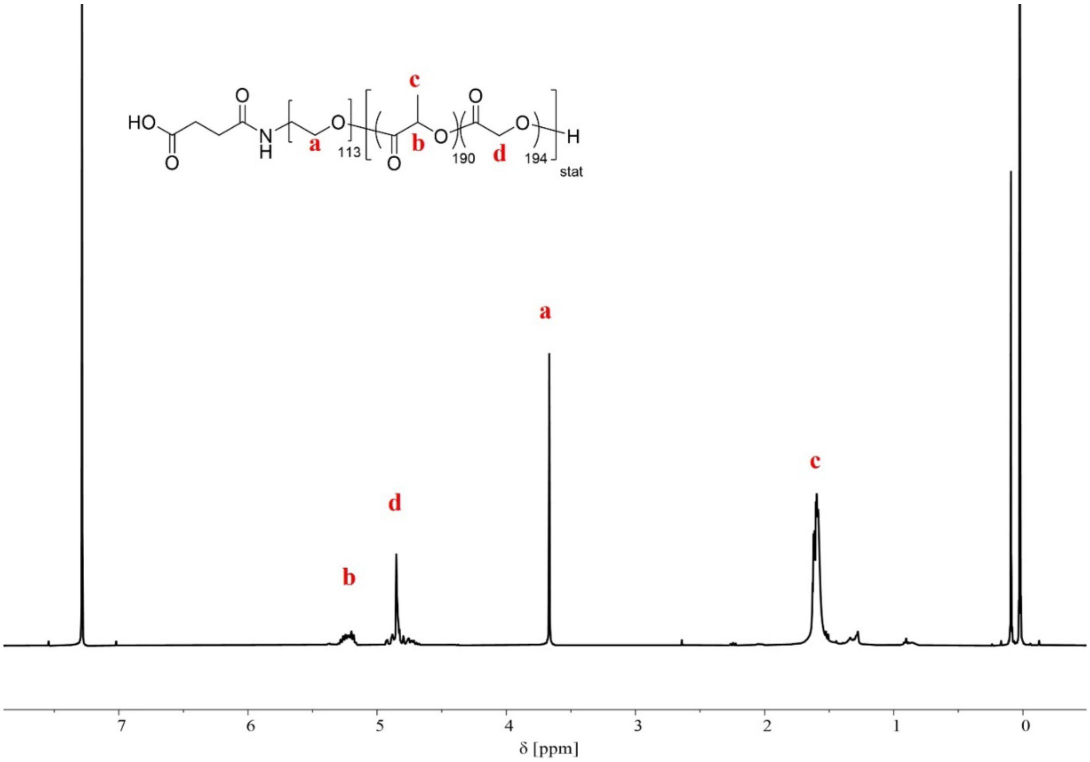

Figure S10: ^{1}H NMR spectrum (400 MHz, $CDCl_3$) of PLGA-PEG-COOH

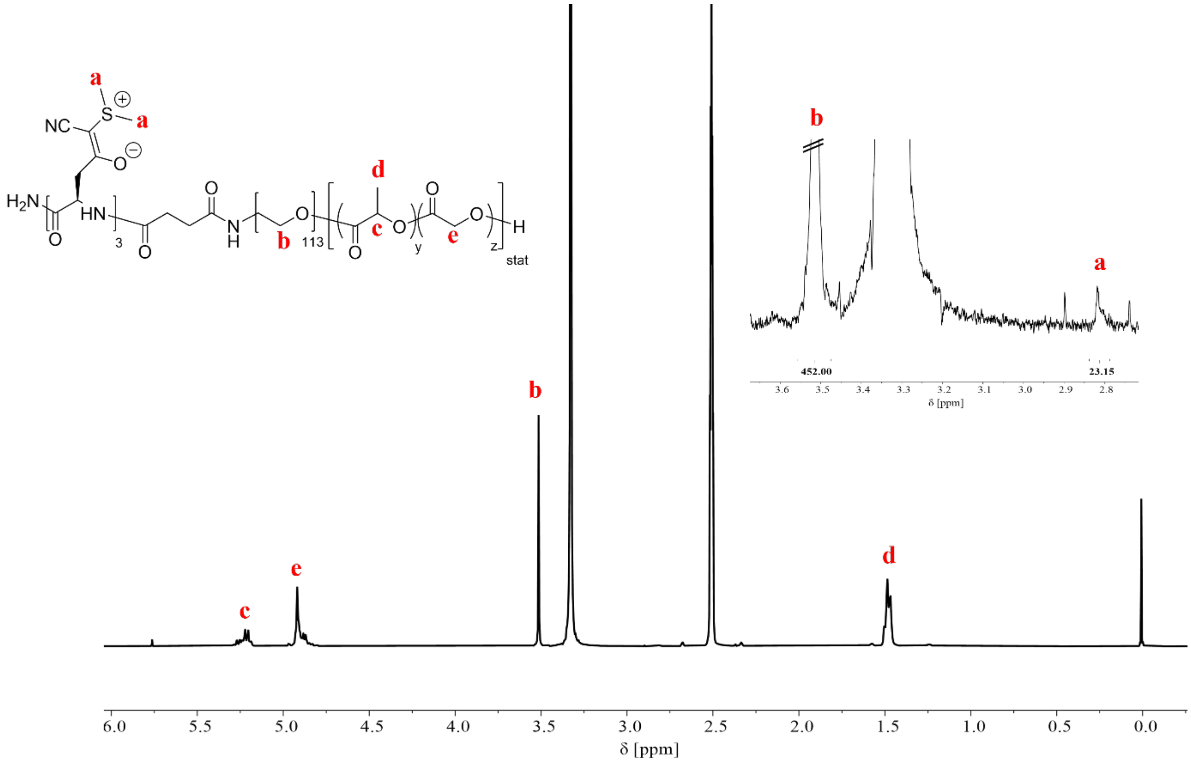

Figure S11: ^{1}H NMR spectrum (400 MHz, DMSO-d_6) of PLGA-PEG-3Asp(SY).

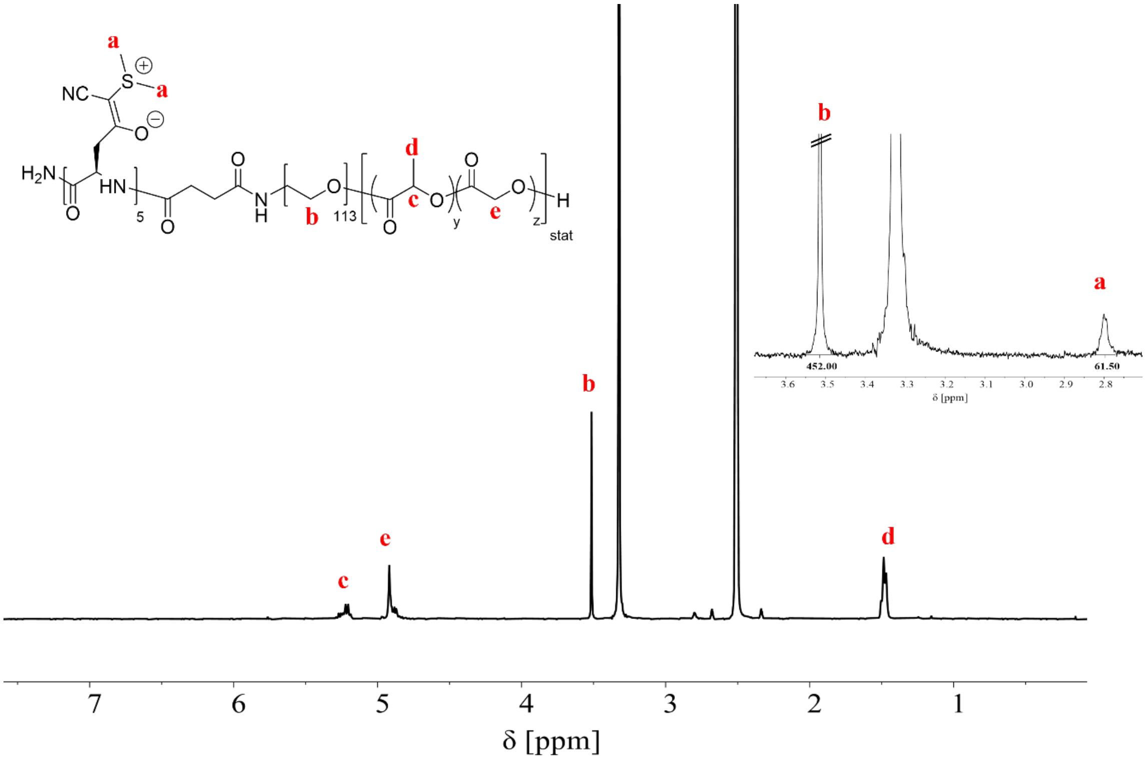

Figure S12: ^{1}H NMR spectrum (400 MHz, DMSO-d_6) of PLGA-PEG-5Asp(SY).

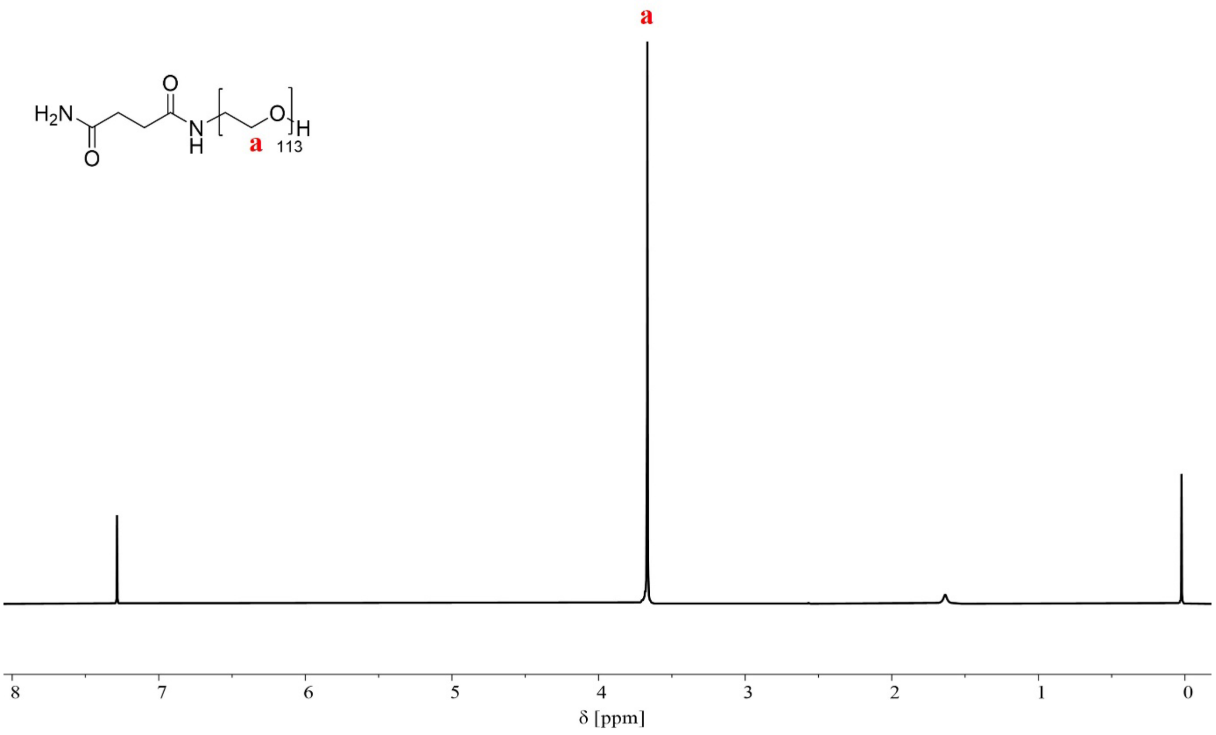

Figure S13: ^{1}H NMR spectrum (400 MHz, $CDCl_3$) of HO-PEG-$CONH_2$

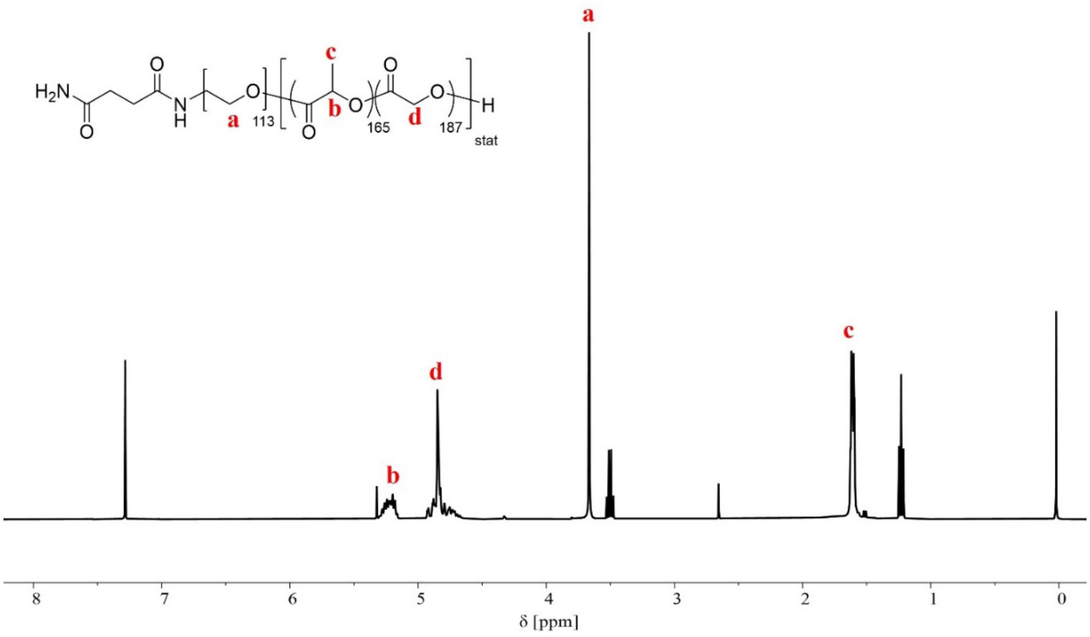

Figure S14: ^{1}H NMR spectrum (400 MHz, $CDCl_3$) of PLGA-PEG-$CONH_2$.

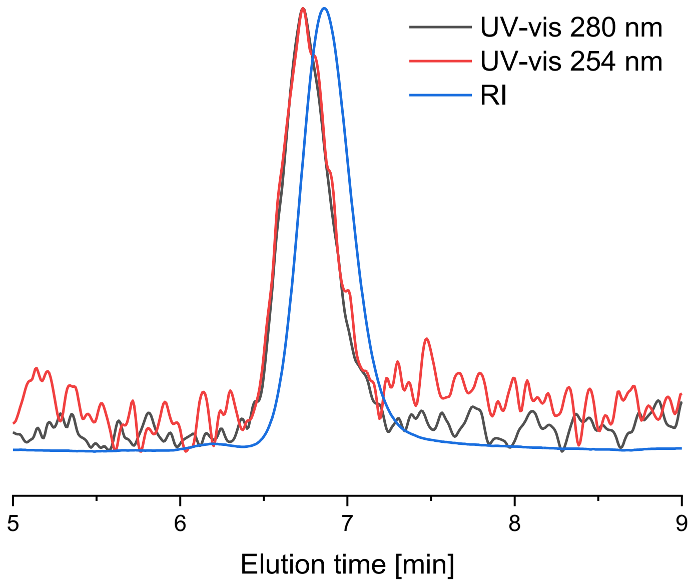

Figure S15: Gel permeation chromatography trace of purified PEG-3Asp(SY).

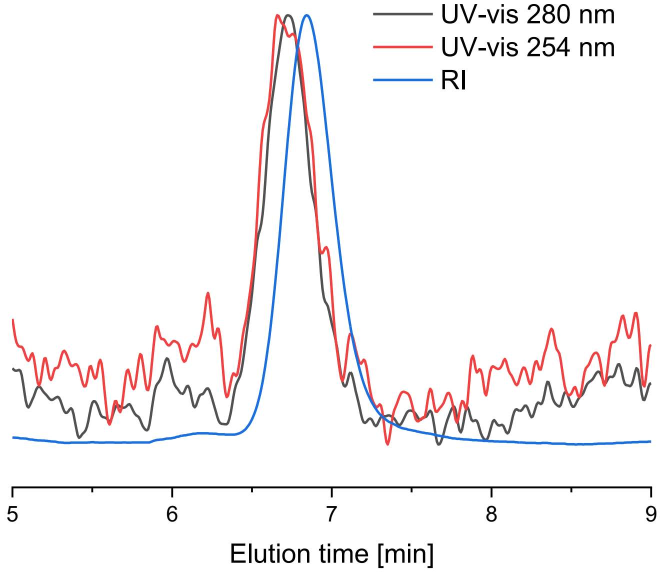

Figure S16: Gel permeation chromatography trace of purified PEG-5Asp(SY).

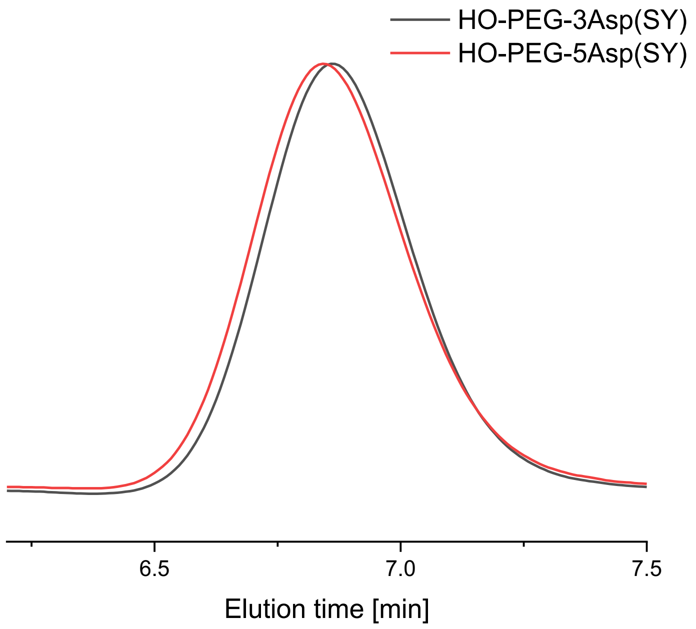

Figure S17: Gel permeation chromatography overlay traces of PEG-3Asp(SY) and PEG-5Asp(SY).

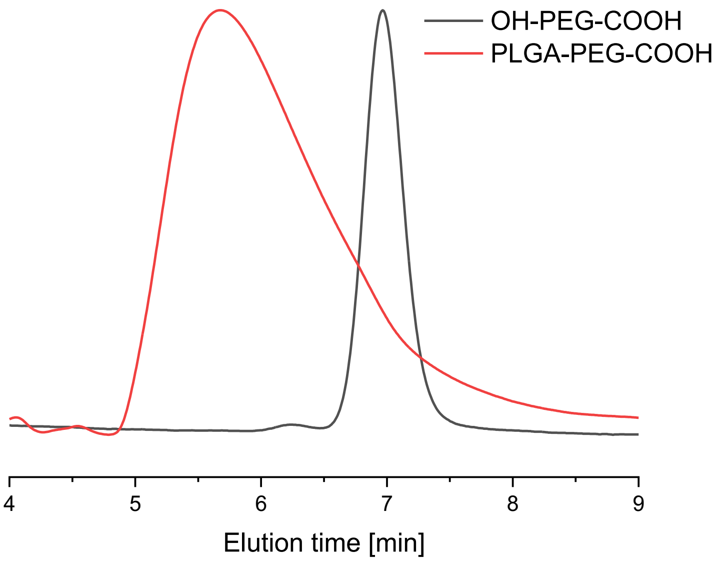

Figure S18: Gel permeation chromatography overlay traces of HO-PEG-COOH macroinitiator and PLGA-PEG-COOH

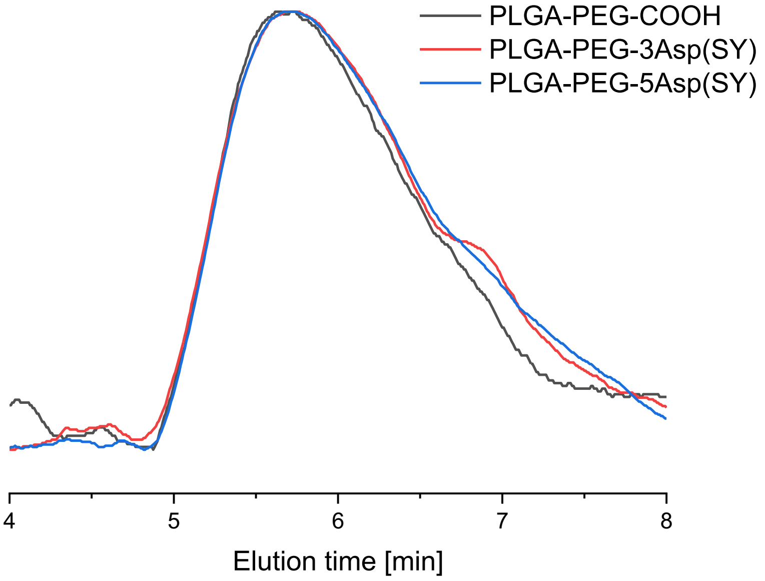

Figure S19: Gel permeation chromatography overlay traces PLGA-PEG-COOH, PLGA-PEG-3Asp(SY), and PLGA-PEG-5Asp(SY).

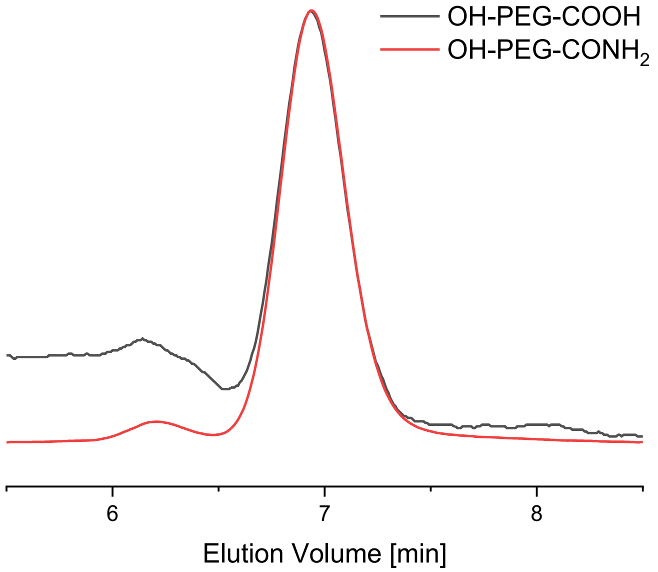

Figure S20: Gel permeation chromatography overlay traces of HO-PEG-COOH and HO-PEG-$CONH_2$.

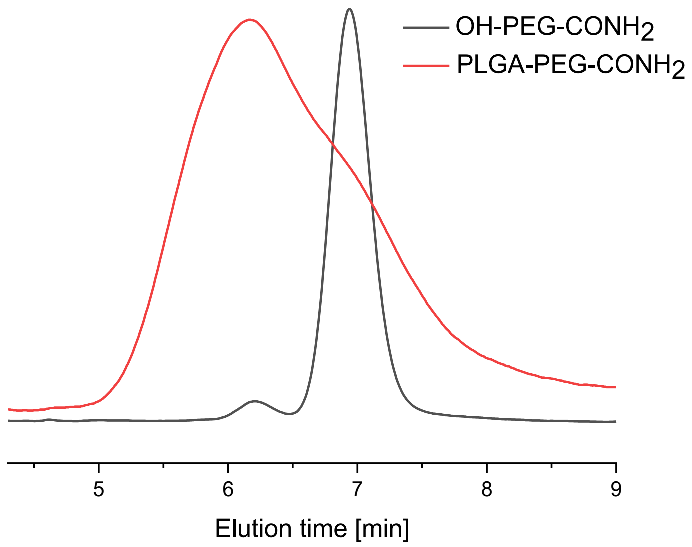

Figure S21: Gel permeation chromatography overlay traces of HO-PEG-CONH$_2$ macroinitiator and PLGA-PEG-CONH$_2$.

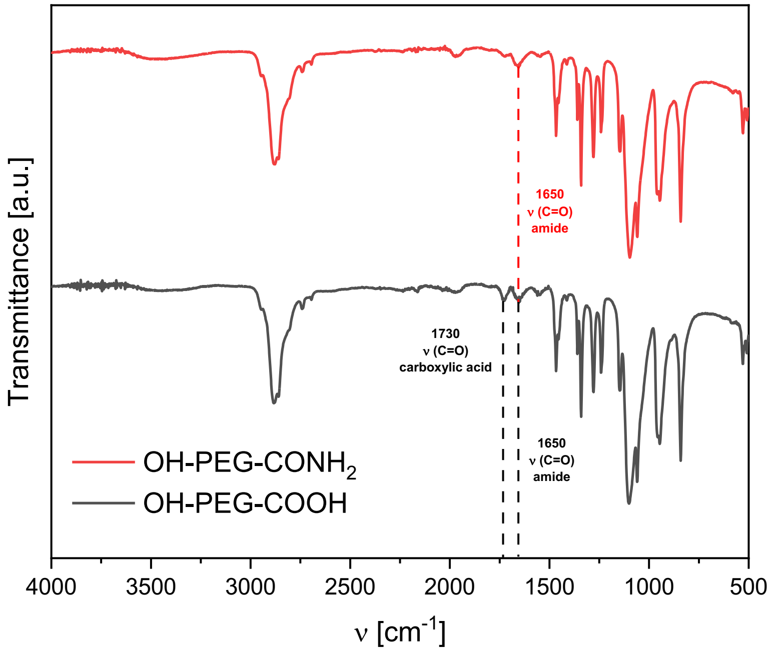

Figure S22: FTIR overlay spectra of HO-PEG-COOH and HO-PEG-CONH$_2$.

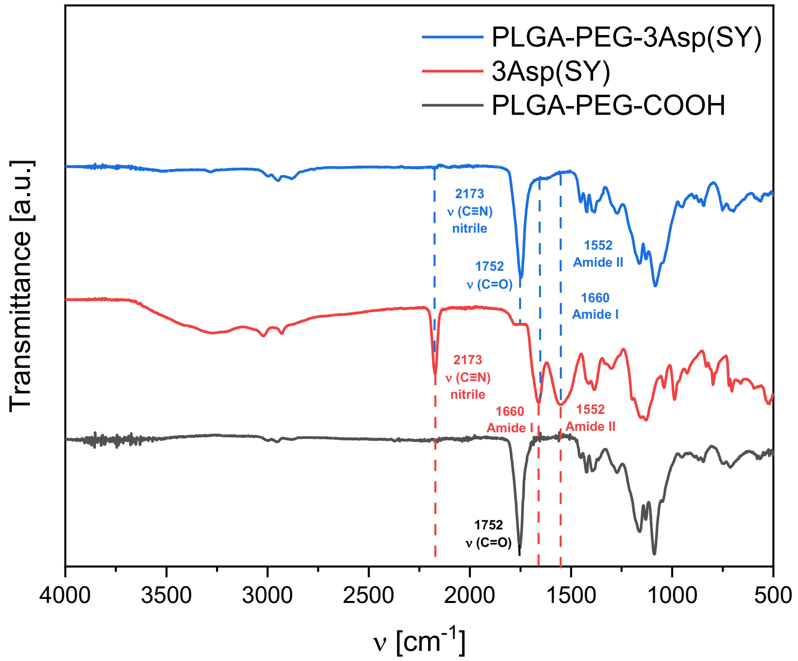

Figure S23: FTIR overlay spectra of PLGA-PEG-COOH, 3Asp(SY), and PLGA-PEG-3Asp(SY).

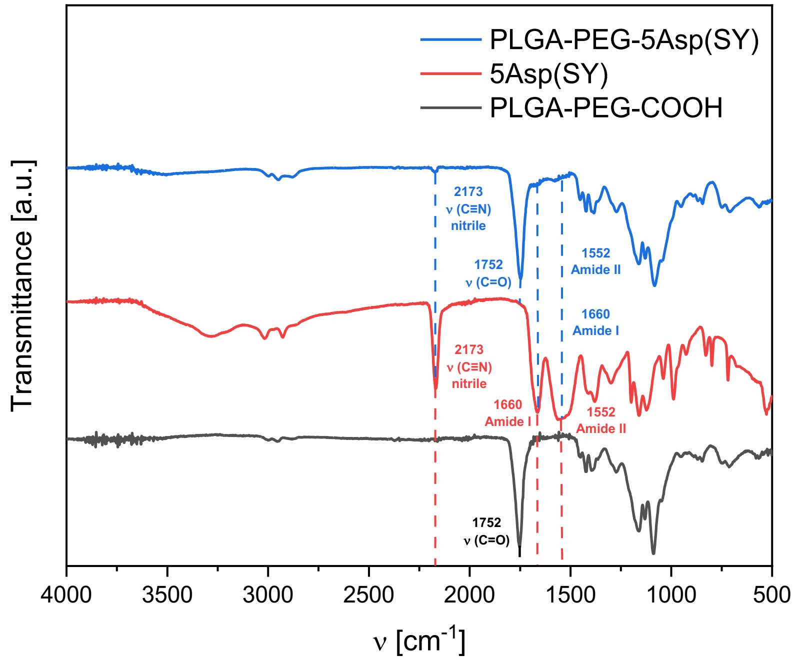

Figure S24: FTIR overlay spectra of PLGA-PEG-COOH, 5Asp(SY), and PLGA-PEG-5Asp(SY).

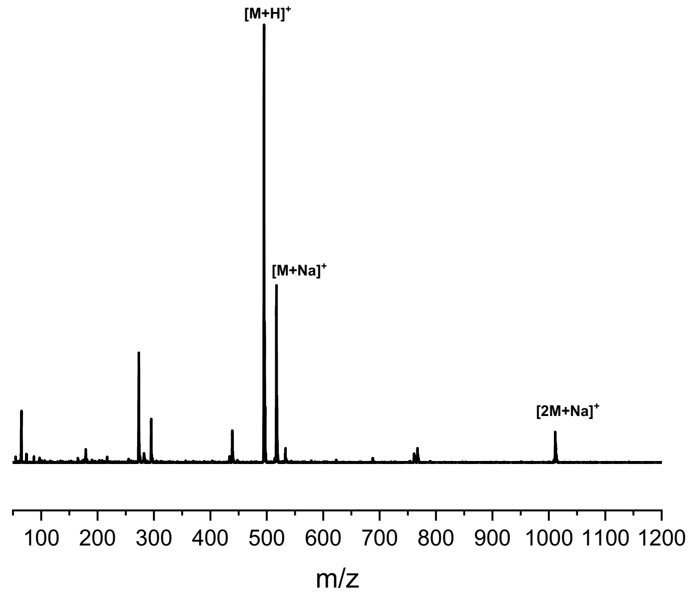

Figure S25: ESI-MS spectrum of Fmoc-Asp(SY)-OtBu.

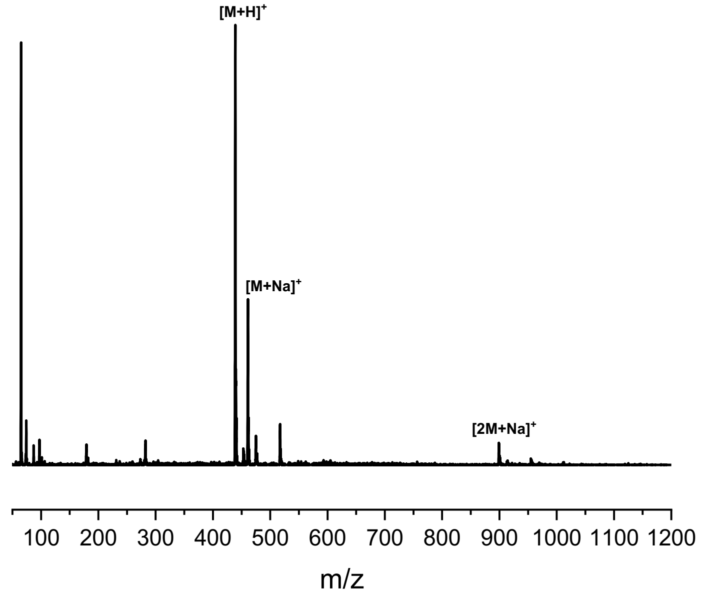

Figure S26: ESI-MS spectrum of Fmoc-Asp(SY)-OH.

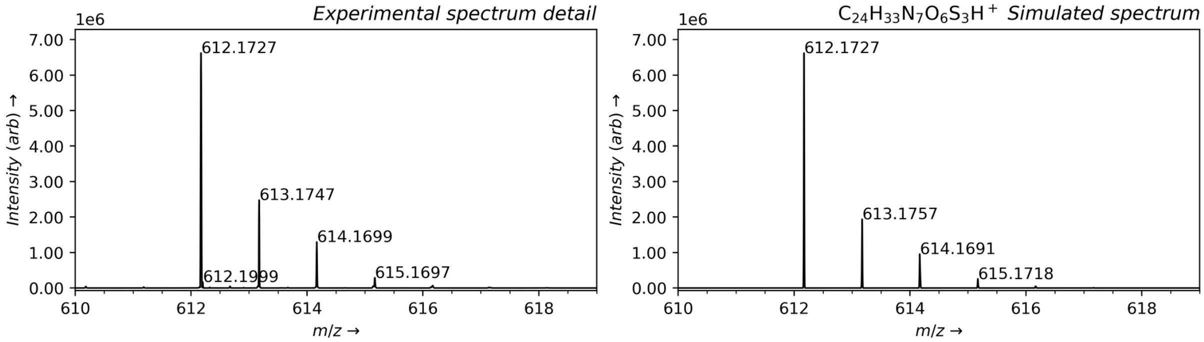

Figure S27: HRMS of 3Asp(SY).

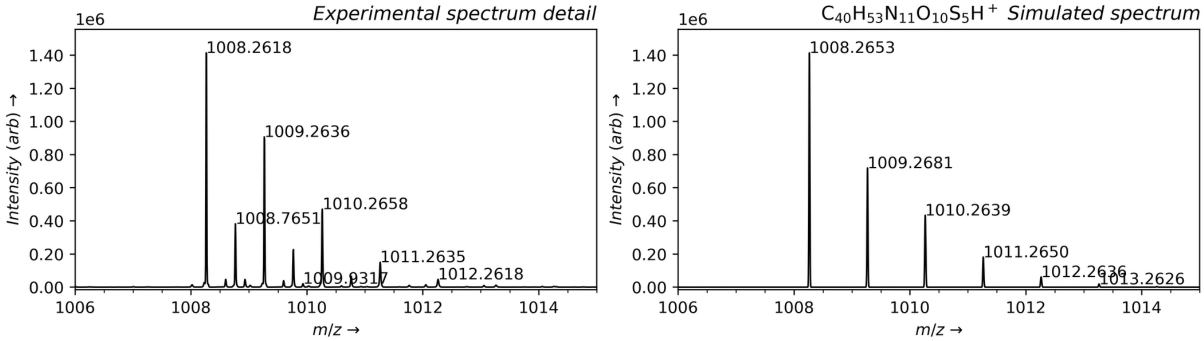

Figure 28: HRMS of 5Asp(SY).

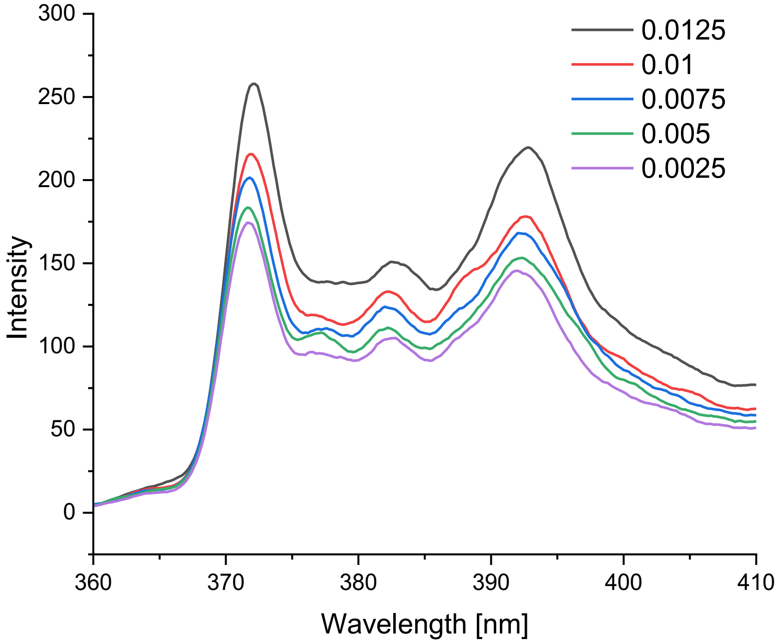

Figure S29: Fluorescence emission spectra of pyrene (6 x 10^{-7} M) in aqueous media of the PLGA-*b*-PEG-3Asp(SY).

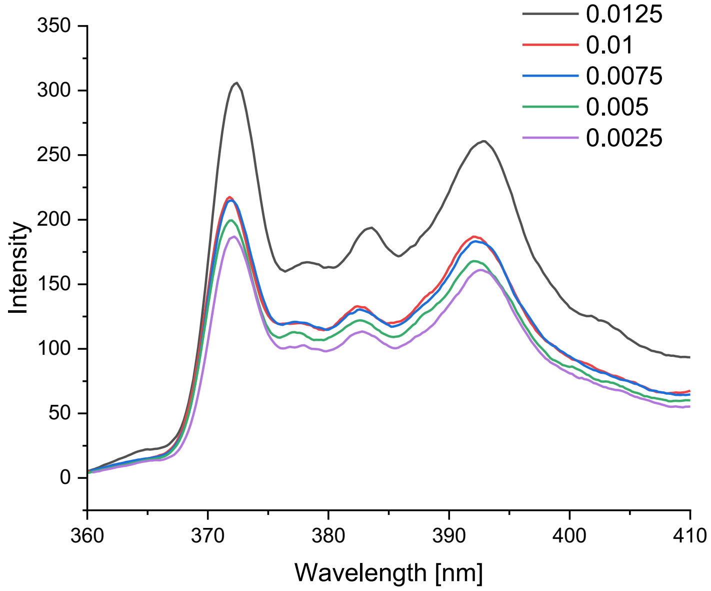

Figure S30: Fluorescence emission spectra of pyrene (6 x 10^{-7} M) in aqueous media of the PLGA-*b*-PEG-5Asp(SY).

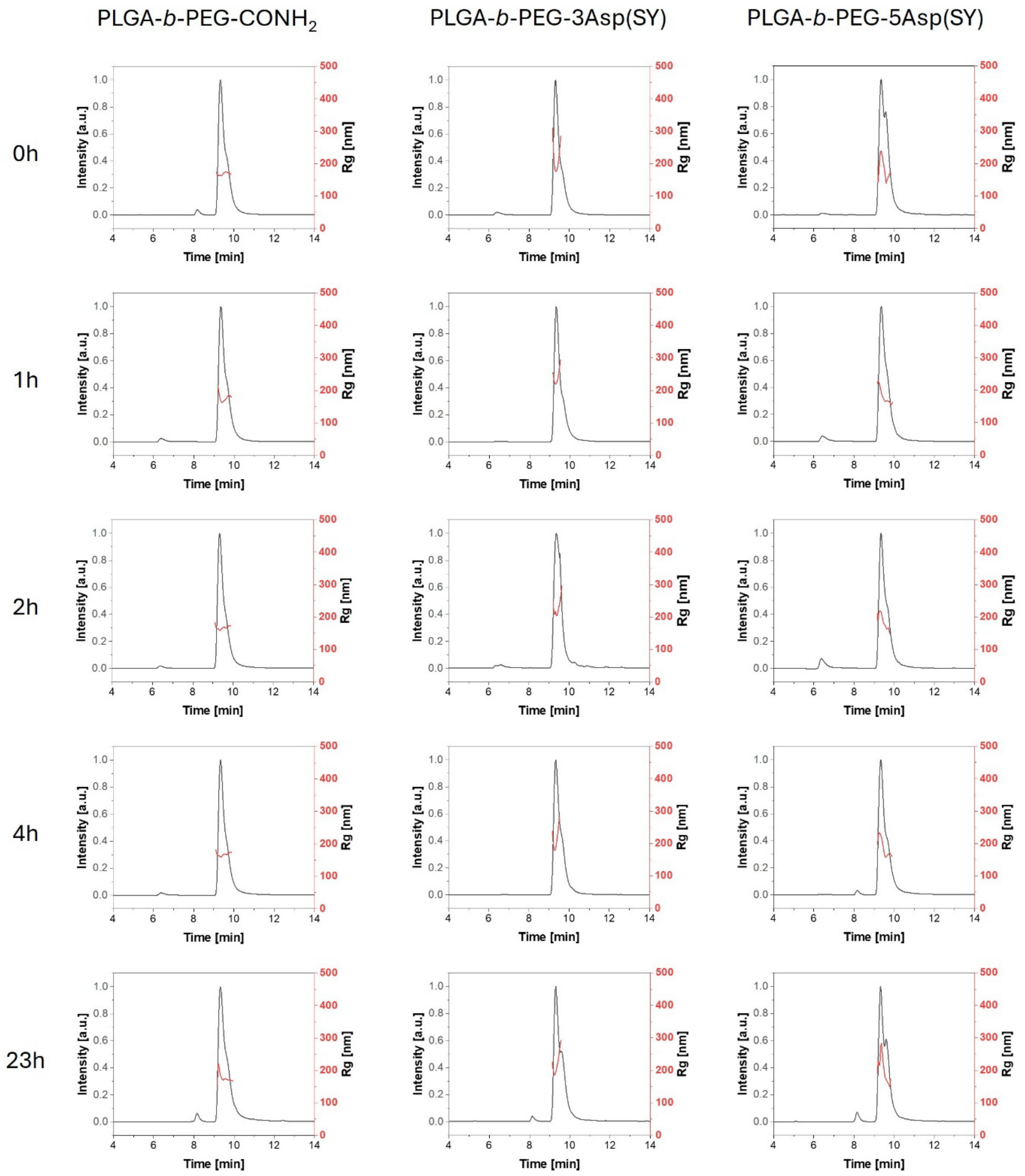

Figure S31: Chromatograms (dark grey) and rms radius (red) of samples PLGA-*b*-PEG-CONH$_2$, PLGA-*b*-3Asp(SY), and PLGA-*b*-5Asp (SY) at times 0h, 1h, 2h, 4h, and 24h without enzyme treatment.

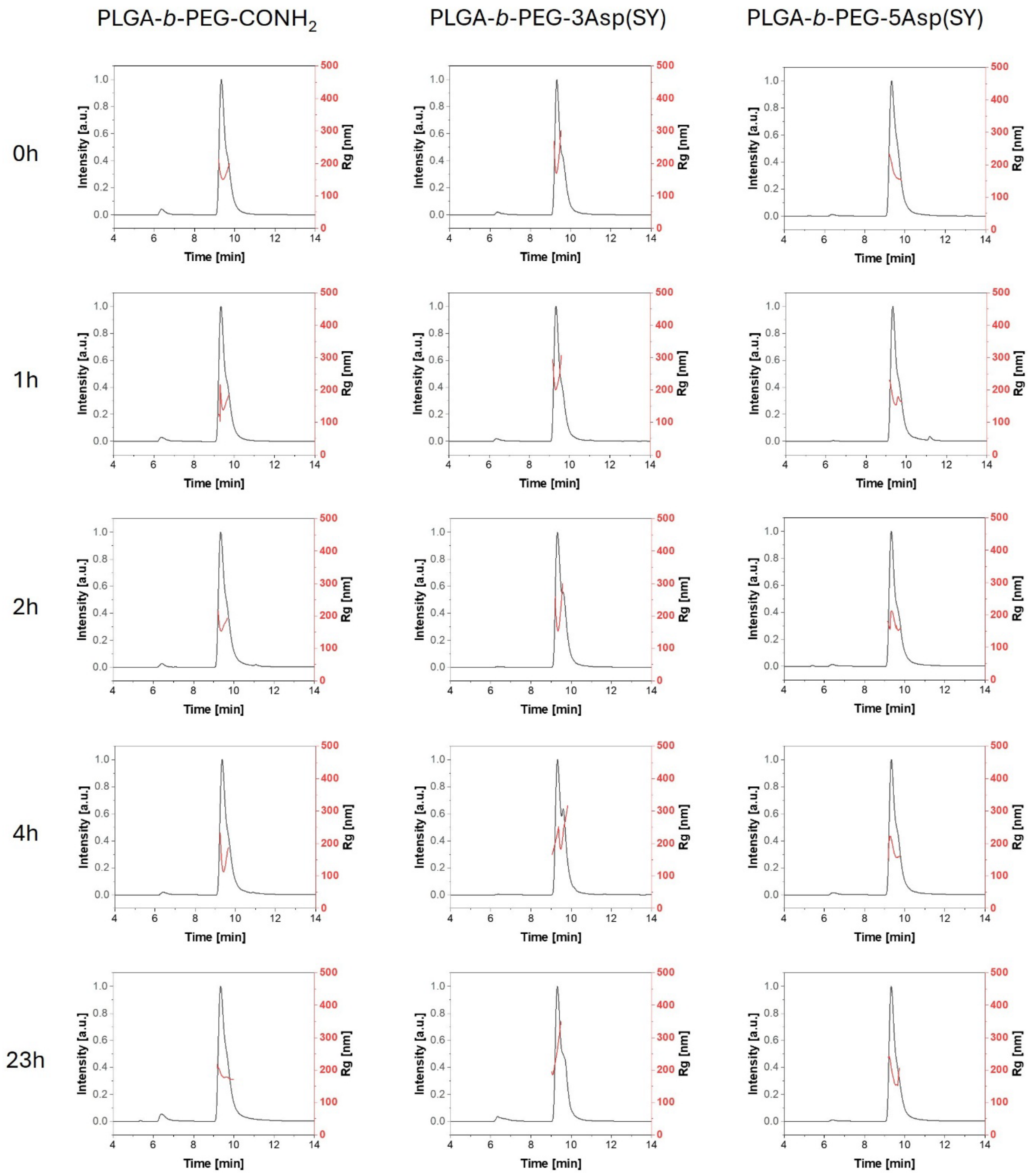

Figure S32: Chromatograms (dark grey) and rms radius (red) of samples PLGA-*b*-PEG-CONH$_2$, PLGA-*b*-3Asp(SY), and PLGA-*b*-5Asp (SY) at times 0h, 1h, 2h, 4h, and 24h with BSA treatment.

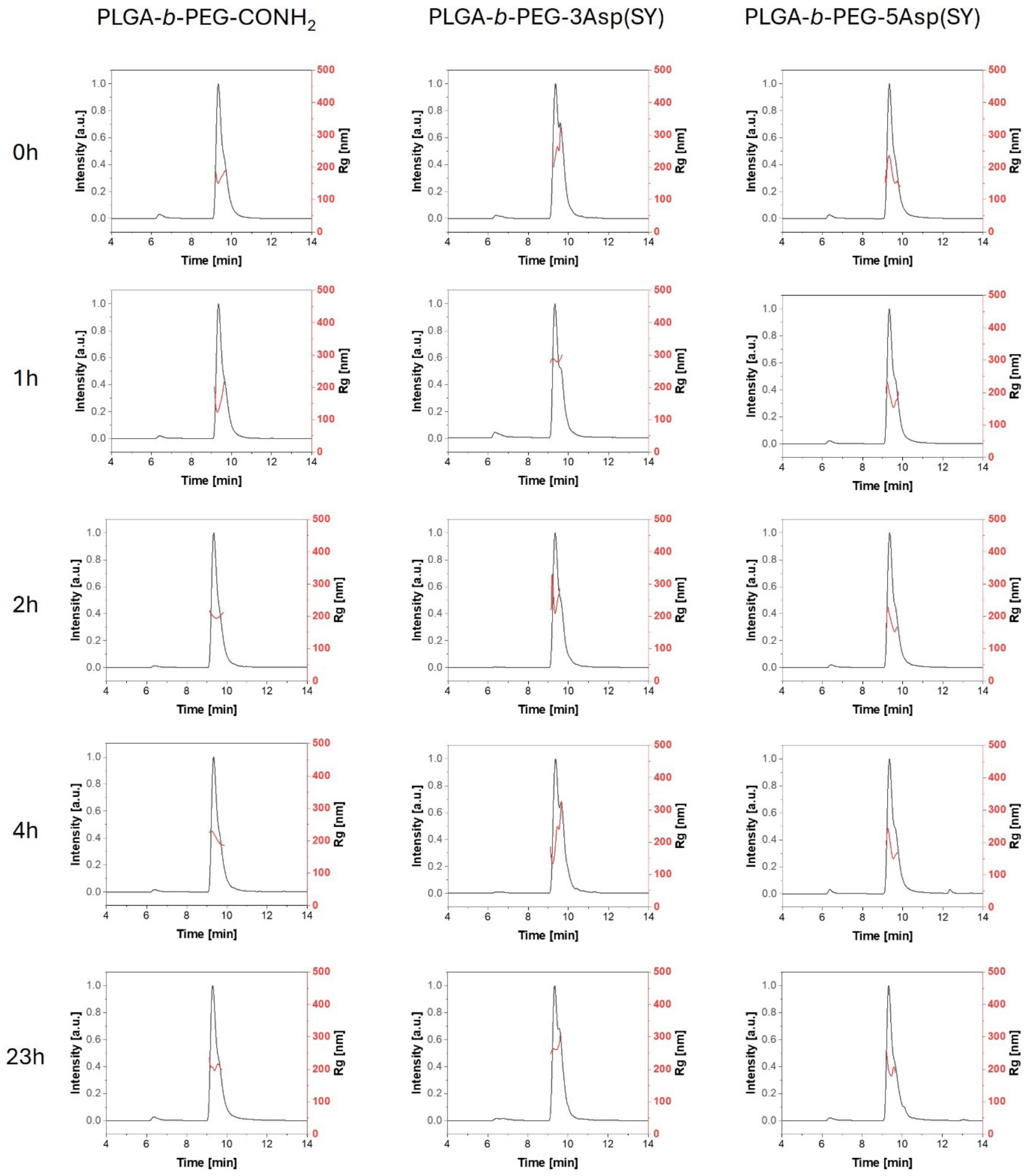

Figure S33: Chromatograms (dark grey) and rms radius (red) of samples PLGA-*b*-PEG-CONH$_2$, PLGA-*b*-3Asp(SY), and PLGA-*b*-5Asp (SY) at times 0h, 1h, 2h, 4h, and 24h with Lysozyme treatment.

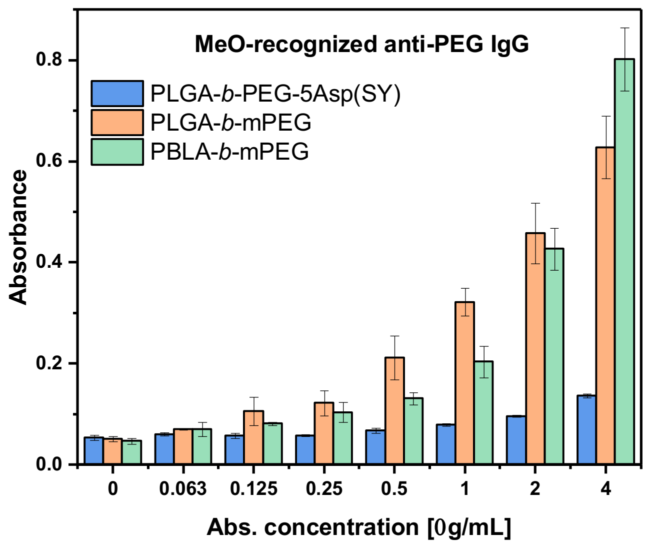

Figure S34: Bindings of terminal methoxy specific anti-PEG IgG to different terminal types of PEGs by indirect ELISA. Different terminal types of PEGs (20 µg/mL in ethanol/H_2O) were used for plate coating at 4 °C for overnight. Bound terminal methoxy specific anti-PEG IgG was detected by HRP-conjugated anti-mouse IgG (0.0125 µg/mL).

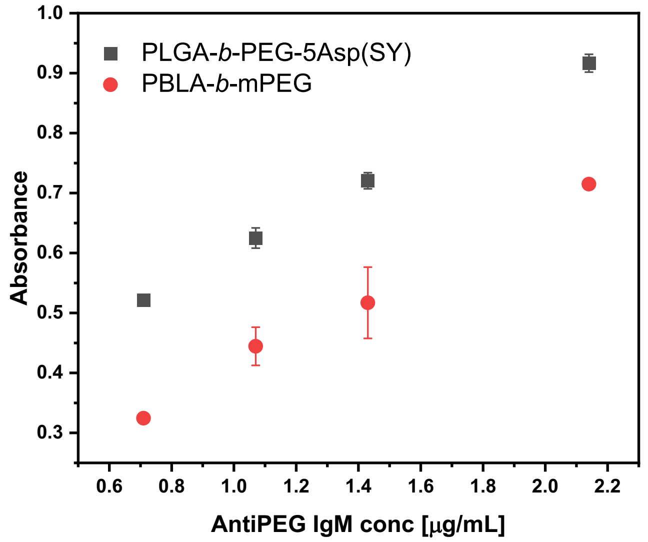

Figure S35: Calibration line for sera experiments with polymers. Standard curves of main chain specific anti-PEG IgM to different terminal types of PEGs by indirect ELISA. Polymers (20 µg/mL in ethanol/H_2O) were used for plate coating at 4 °C for overnight.

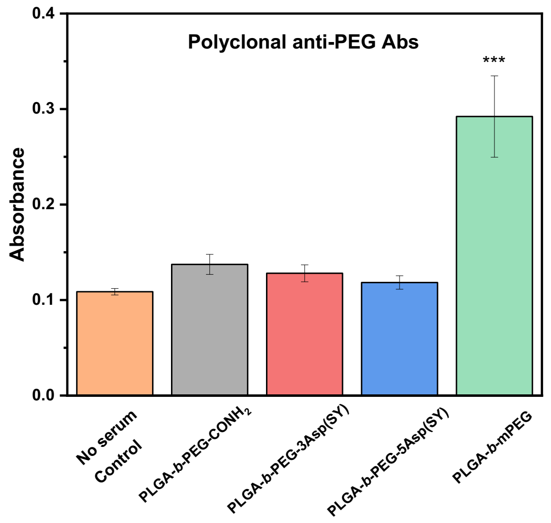

Figure S36: Bindings of polyclonal anti-PEG IgM in sera were confirmed by indirect ELISA. Polymers (20 μg/mL in ethanol/H_2O) were used for plate coating at 4 °C for overnight. A 50 times saline diluted sera containing anti-PEG IgM were added to different terminal types of PEG-coated plates. HRP-conjugated anti-mouse IgM (0.0125 μg/mL) was used as a detection antibody.

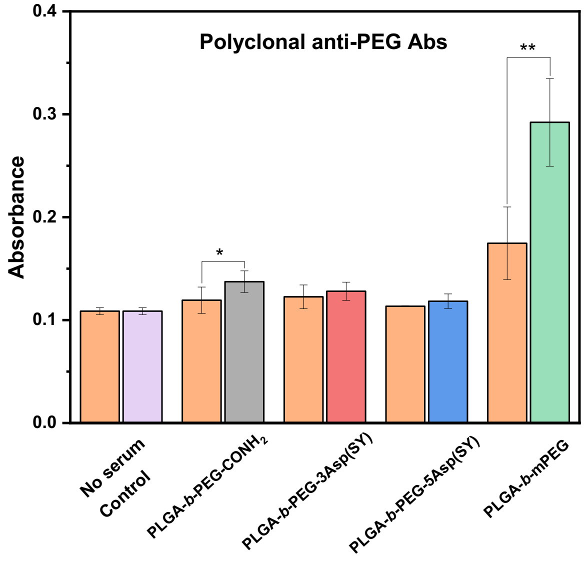

Figure S37: Bindings of polyclonal anti-PEG IgM in sera were confirmed by indirect ELISA; here depicted with the corresponding controls using only sera. Polymers (20 μg/mL in ethanol/H_2O) were used for plate coating at 4 °C for overnight. A 50 times saline diluted sera containing anti-PEG IgM or sera containing no anti-PEG IgM were added to different terminal types of PEG-coated plates. HRP-conjugated anti-mouse IgM (0.0125 μg/mL) was used as a detection antibody.

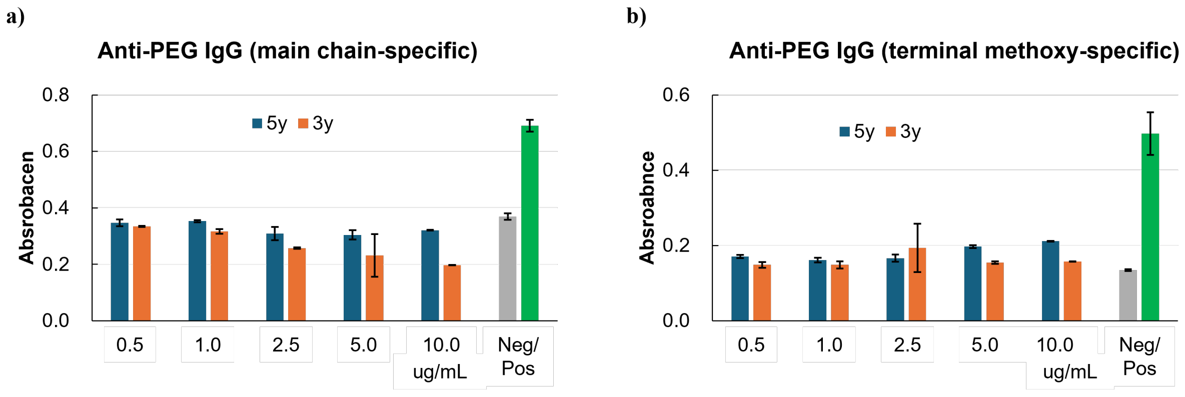

Figure S38: Bindings of a) main chain specific anti-PEG IgG and b) terminal methoxy-specific anti-PEG IgG to PLGA-b-PEG-5Asp(SY) (5SY) or PLGA-b-PEG-3Asp(SY) by sandwich ELISA .

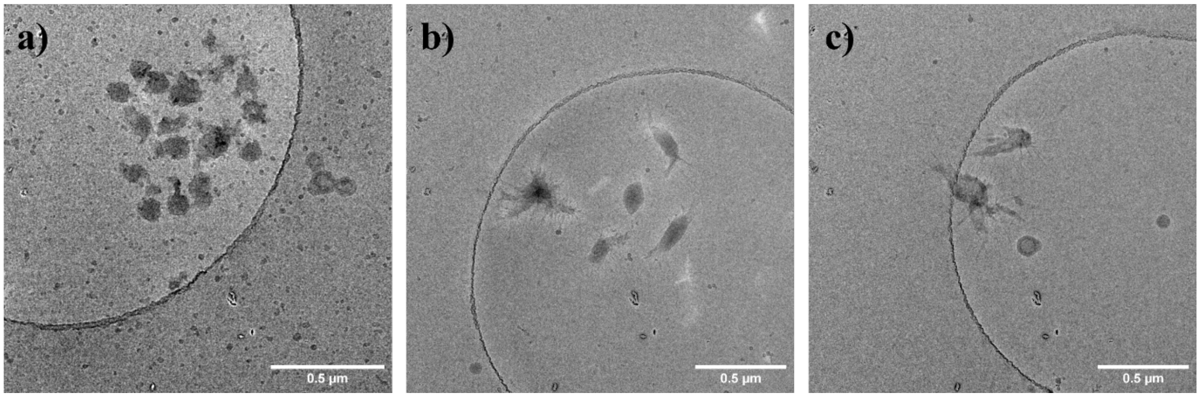

Figure S39. CryoTEM images of a) PLGA-*b*-PEG-$CONH_2$ NPs, b) PLGA-*b*-PEG-3Asp(SY) NPs, and c) PLGA-*b*-PEG-5Asp(SY) NPs

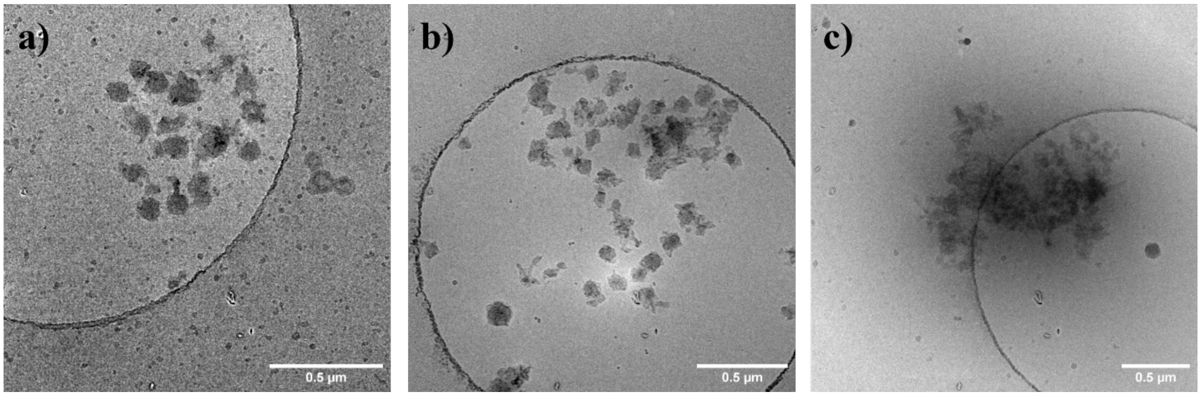

Figure S40. CryoTEM images of PLGA-*b*-PEG-$CONH_2$ NPs at a) t = 0 h, b) t = 2 h, and c) t = 24 h After incubation in DMEM with 10% FBS at 37 °C

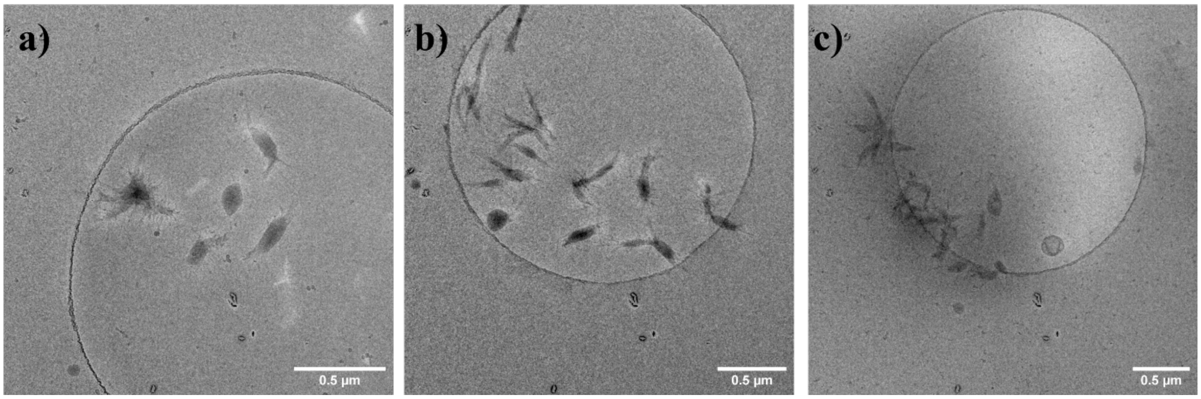

Figure S41. CryoTEM images of PLGA-*b*-PEG-3Asp(SY) NPs at a) t = 0 h, b) t = 2 h, and c) t = 24 h After incubation in DMEM with 10% FBS at 37 °C

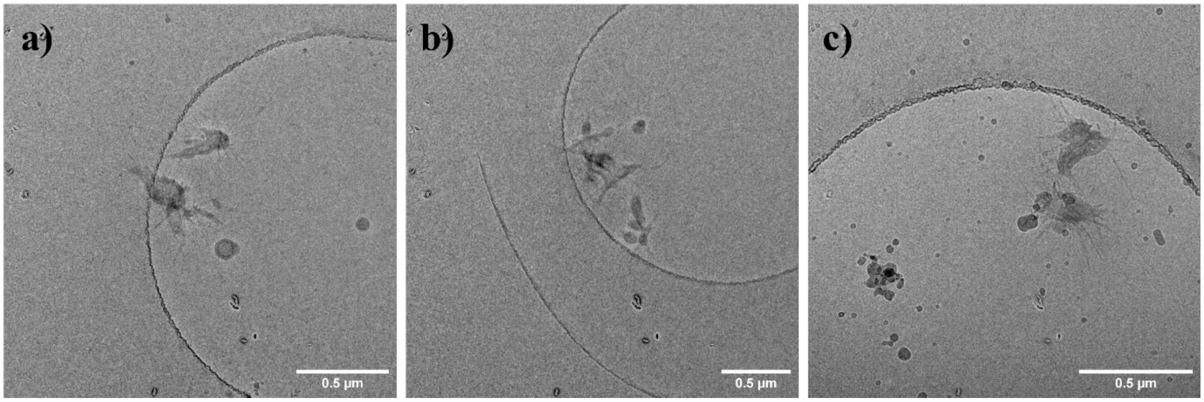

Figure S42. CryoTEM images of PLGA-*b*-PEG-3Asp(SY) NPs at a) t = 0 h, b) t = 2 h, and c) t = 24 h After incubation in DMEM with 10% FBS at 37 °C

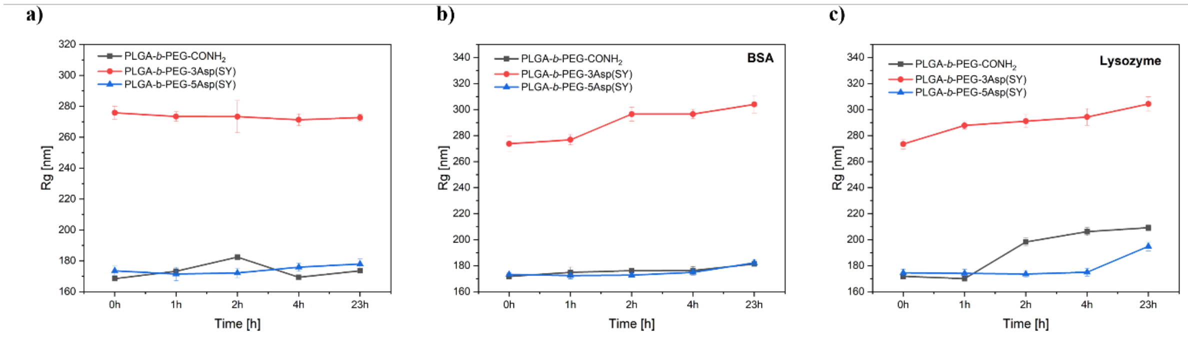

Figure S43. Rg values of PLGA-*b*-PEG-CONH$_2$, PLGA-*b*-PEG-3Asp(SY), and PLGA-*b*-PEG-5Asp(SY) nanoparticles under a) no treatment, b) BSA treatment and c) lysozyme treatment at time points 0h, 1h, 2h, 4h, and 23h

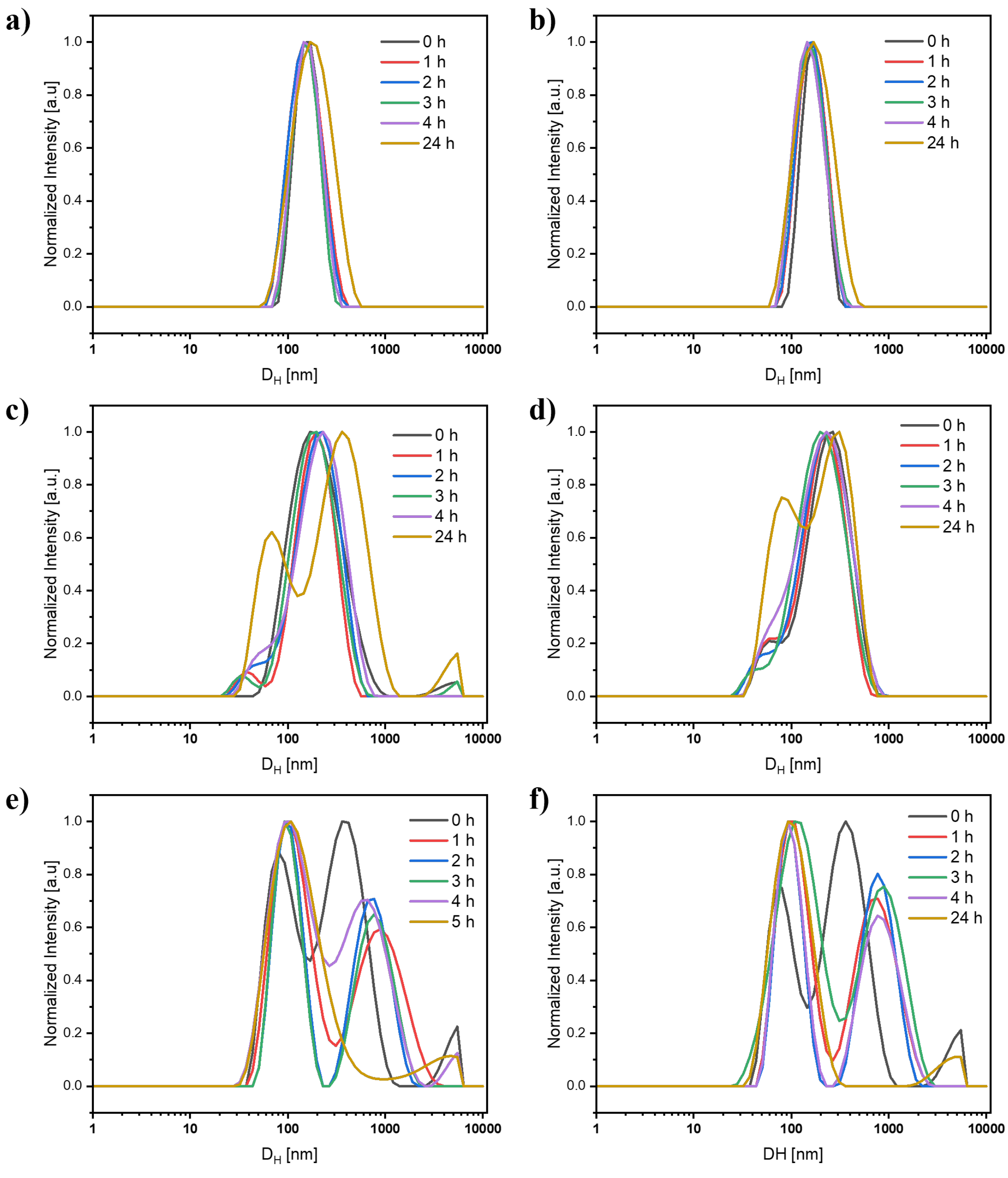

Figure S44. Hydrodynamic diameter of PLGA-*b*-PEG-CONH2 under a) BSA treatment, b) Lysozyme treatment. Hydrodynamic diameter of PLGA-*b*-PEG-3Asp(SY) under c) BSA treatment, d) Lysozyme treatment. Hydrodynamic diameter of PLGA-*b*-PEG-5Asp(SY) under e) BSA treatment, f) Lysozyme treatment.Particles were incubated in DPBS buffer with the respective proteins to give a final concentration of 0.5 mg/mL.

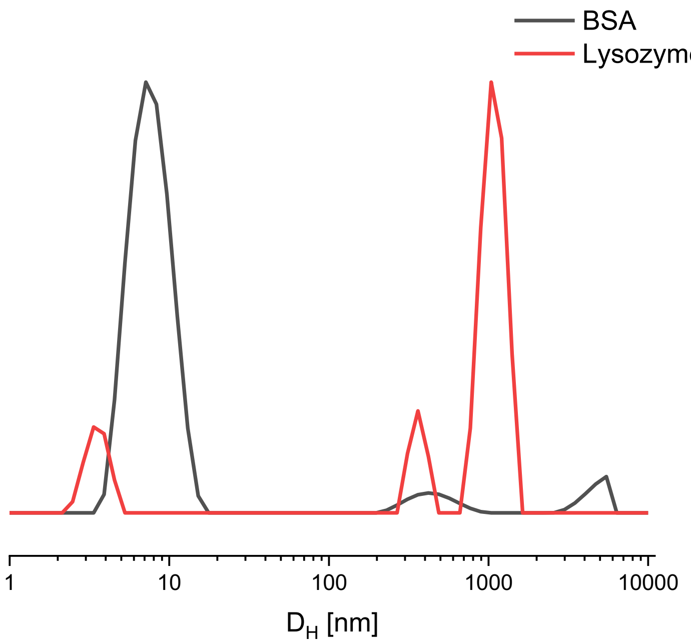

Figure S45. Hydrodynamic diameter of BSA (black) and Lysozyme (red) after 24 h incubation in DPBS buffer at 37 ° at a concentration of 0.5 mg/mL.

Supplementary tables

Table S1. Characterization data of the commercially available HO-PEG-COOH and the synthesized polymeric systems.

Polymer	M_n[a]	M_n[b]	Đ[a]	DP[b]
	[g/mol]	[g/mol]		[PEG:PLA:PGA]
HO-PEG-COOH	8939	4972	1.01	44:0:0
PLGA-b-PEG-COOH	21929	29923	1.35	44:95:97
HO-PEG-NH_2	9329	4972	1.01	44:0:0
PLGA-*b*-PEG-$CONH_2$	18958	27643	1.56	44:82.5:93.5
HO-PEG-3Asp(SY)	9855	5583	1.02	44:0:0
HO-PEG-5Asp(SY)	9999	5979	1.02	44:0:0
PLGA-*b*-PEG-3Asp(SY)	17624	30534	1.60	44:95:97
PLGA-*b*-PEG-5Asp(SY)	17585	30930	1.56	44:95:97

[a] GPC (DMAc, RI-detection, PS calibration).
[b] ^{1}H NMR ($CDCl_3$)

Table S2: Gyroscopic radius (Rg), hydrodynamic radius (Rh), and centre of mass (Rg/Rh) of nanoparticles PLGA-b-PEG-$CONH_2$, PLGA-b-PEG-3Asp(SY), and PLGA-b-PEG-5Asp(SY) at time points 0h, 1h, 2h, 4h, and 23h measured by FFF-MALS.

		Time	Rg (nm)	St dev	Rh (nm)	St dev	Rg/Rh	St dev
PLGA-*b*-PEG-$CONH_2$	Particle	0h	168.5	1.348	170	1.530	0.99	0.006
		1h	173.3	1.040	170	0.850	1.02	0.005
		2h	182.4	1.459	170	1.190	1.07	0.007
		4h	169.4	1.525	170	1.360	1.00	0.007
		23h	173.6	0.694	170	0.850	1.02	0.003
	BSA	0h	171.8	0.172	170	0.510	1.01	0.001
		1h	174.9	4.023	170	2.380	1.03	0.019
		2h	176.2	0.529	170	0.170	1.04	0.003
		4h	176.3	2.997	170	3.740	1.04	0.014
		23h	181.4	0.726	170	1.360	1.07	0.003
	Lysozyme	0h	171.9	0.688	170	1.020	1.01	0.003
		1h	170.1	2.041	170	1.530	1.00	0.010
		2h	198.3	3.173	170	3.910	1.17	0.015
		4h	206.2	3.093	170	0.850	1.21	0.015
		23h	209.2	2.301	170	3.060	1.23	0.011
PLGA-b-PEG-3Asp(SY)	Particle	0h	275.8	4.137	267	2.670	1.03	0.013
		1h	273.4	3.007	267	1.869	1.02	0.009
		2h	273.3	10.385	267	8.277	1.02	0.032
		4h	271.3	3.798	267	5.607	1.02	0.012
		23h	272.7	2.182	267	0.534	1.02	0.007
	BSA	0h	273.7	5.748	267	3.204	1.03	0.018
		1h	276.8	3.875	267	5.340	1.04	0.012
		2h	296.5	5.337	267	2.670	1.11	0.016
		4h	296.5	3.558	267	2.136	1.11	0.011
		23h	304.0	6.688	267	4.272	1.14	0.020
	Lysozyme	0h	273.5	3.829	267	4.806	1.02	0.012
		1h	287.8	2.878	267	3.204	1.08	0.009
		2h	291.1	4.949	267	3.471	1.09	0.015
		4h	294.3	6.475	267	6.141	1.10	0.020
		23h	304.3	5.477	267	2.937	1.14	0.017
PLGA-b-PEG-5Asp(SY)	Particle	0h	173.6	2.951	170	2.380	1.02	0.014
		1h	171.5	4.288	170	3.740	1.01	0.021
		2h	172.2	3.100	170	3.570	1.01	0.015
		4h	175.9	2.639	170	2.720	1.03	0.013
		23h	178.0	3.204	170	2.720	1.05	0.015
	BSA	0h	173.3	1.386	170	0.680	1.02	0.007

		1h	172.4	2.586	170	1.530	1.01	0.012
		2h	172.8	1.901	170	2.040	1.02	0.009
		4h	174.9	2.274	170	1.190	1.03	0.011
		23h	182.2	2.186	170	1.530	1.07	0.011
	Lysozyme	0h	174.6	2.794	170	1.700	1.03	0.013
		1h	174.2	3.136	170	1.190	1.02	0.015
		2h	173.6	2.604	170	2.210	1.02	0.013
		4h	175.0	2.975	170	2.380	1.03	0.014
		23h	194.9	3.508	170	4.420	1.15	0.017

References

1 C. T. Lollar, K. M. Krenek, K. J. Bruemmer, A. R. Lippert, *Organic & Biomolecular Chemistry,* 2013, ***12***, 406-409.

2 K. Neumann, J. Farnung, S. Baldauf, J. W. Bode, *Nature communications,* 2020, ***11***, 982.

3 M. Ashjari, S. Khoee, A. R. Mahdavian, R. Rahmatolahzadeh, *Journal of materials science: Materials in medicine,* 2012, ***23***, 943-953.
